# Immunodominant extracellular loops of *Treponema pallidum* FadL outer membrane proteins elicit antibodies with opsonic and growth-inhibitory activities

**DOI:** 10.1371/journal.ppat.1012443

**Published:** 2024-12-23

**Authors:** Kristina N. Delgado, Melissa J. Caimano, Isabel C. Orbe, Crystal F. Vicente, Carson J. La Vake, André A. Grassmann, M. Anthony Moody, Justin D. Radolf, Kelly L. Hawley

**Affiliations:** 1 Department of Medicine, UConn Health, Farmington, Connecticut, United States of America; 2 Department of Pediatrics, UConn Health, Farmington, Connecticut, United States of America; 3 Department of Molecular Biology and Biophysics, UConn Health, Farmington, Connecticut, United States of America; 4 Department of Research, Connecticut Children’s Research Institute, Hartford, Connecticut, United States of America; 5 Duke Human Vaccine Institute, Durham, North Carolina, United States of America; 6 Department of Pediatrics, Duke University Medical Center, Durham, North Carolina, United States of America; 7 Department of Immunology, Duke University Medical Center, Durham, North Carolina, United States of America; 8 Department of Immunology, UConn Health, Farmington, Connecticut, United States of America; 9 Department of Genetics and Genome Sciences, UConn Health, Farmington, Connecticut, United States of America; 10 Division of Infectious Diseases and Immunology, Connecticut Children’s, Hartford, Connecticut, United States of America; Université de Reims Champagne-Ardenne: Universite de Reims Champagne-Ardenne, FRANCE

## Abstract

The global resurgence of syphilis has created a potent stimulus for vaccine development. To identify potentially protective antibodies against *Treponema pallidum* (*TPA*), we used *Pyrococcus furiosus* thioredoxin (*Pf*Trx) to display extracellular loops (ECLs) from three *TPA* outer membrane protein families (outer membrane factors for efflux pumps, eight-stranded β-barrels, and FadLs) to assess their reactivity with immune rabbit serum (IRS). We identified five immunodominant loops from the FadL orthologs TP0856, TP0858 and TP0865 by immunoblotting and ELISA. Rabbits and mice immunized with these five *Pf*Trx constructs produced loop-specific antibodies that promoted opsonophagocytosis of *TPA* by rabbit peritoneal and murine bone marrow-derived macrophages at levels comparable to IRS and mouse syphilitic serum. Heat-inactivated IRS and loop-specific rabbit and mouse antisera also impaired viability, motility, and cellular attachment of spirochetes during *in vitro* cultivation. The results support the use of ECL-based vaccines and suggest that loop-specific antibodies promote spirochete clearance via Fc receptor-independent as well as Fc receptor-dependent mechanisms.

## Author summary

The resurgence of syphilis underscores the critical need for vaccine development against *Treponema pallidum* (*TPA*). Prior research utilizing immune rabbit serum (IRS) suggests that an effective syphilis vaccine should induce “functional” antibodies capable of enhancing the opsonophagocytosis of treponemes by activated macrophages. Structural models of *TPA* outer membrane proteins (OMPs), specifically the extracellular loops (ECLs), guided the identification of potential vaccine candidates. Antigenic analysis with IRS of individual ECLs from three *TPA* OMP families scaffolded onto *Pyrococcus furiosus* thioredoxin (*Pf*Trx) revealed five FadL antigenic loops. Immunization with these immunodominant loop antigens elicited robust ECL-specific antibodies, demonstrating functional opsonic activity in the opsonophagocytosis assays. Furthermore, these antibodies effectively inhibited the growth of *in vitro* cultivated *TPA* in both rabbit and mouse models. Our findings underscore the value of antigenic analysis in identifying promising *TPA* ECL vaccine targets and highlight the multifaceted protective capacity of loop antibodies against *TPA*. This approach also extends to identifying potential OMP vaccinogens in other bacterial pathogens, offering valuable insights for broader vaccine development strategies.

## Introduction

Syphilis is a multistage, sexually transmitted infection caused by the highly invasive and immunoevasive spirochete *Treponema pallidum* subsp. *pallidum* (*TPA*) [1, 2]. Since the start of the current millennium, the disease has undergone a dramatic resurgence in the United States and worldwide even though its causative agent remains exquisitely susceptible to penicillin after more than seven decades of use [1–3]. These alarming trends underscore the urgent need for new control strategies, including vaccines [4, 5]. The rabbit has long been considered the animal model of choice for investigating protective immunity against syphilitic infection [6–8]. Rabbits develop long-lasting immunity to reinfection [6–9], and it is generally believed that deconvolution of protective responses in the rabbit will inform vaccine development for humans. Evidence from the rabbit model [10], supported by subsequent *ex vivo* studies with human syphilitic sera [11–13], has brought to light the importance of macrophage-mediated opsonophagocytosis as a primary mechanism for clearance of *TPA*. Accordingly, it is generally believed that opsonic antibodies for *TPA* can be considered a surrogate for protection [10, 14]. Whether opsonophagocytosis is the sole mechanism for antibody-mediated clearance of *TPA* in humans or animals, however, remains to be determined. Historically, mouse models have not found widespread acceptance in the syphilis field [15, 16]. Nevertheless, *TPA*-infected mice clear the infection and produce antibodies that promote uptake and degradation of spirochetes by bone marrow-derived macrophages (BMDMs) [17–19]. These results suggest that the mouse model has potential utility to expedite selection and evaluation of syphilis vaccine candidates.

Extensive investigation of the molecular architecture of the *TPA* outer membrane (OM) has identified the spirochete’s repertoire of OM proteins (OMPs) as the principal candidate antigens for syphilis vaccine design [20–25]. The *TPA* OMPeome consists of two proteins, BamA (TP0326) and LptD (TP0515), involved in OM biogenesis and four paralogous families involved in importation of nutrients or extrusion of noxious substances across the OM: OM factors (OMFs) for efflux pumps, eight-stranded β-barrels (8SβBs), long-chain fatty acid transporters (FadLs), and *Treponema pallidum* repeat proteins (Tprs) [23, 24]. As in other diderm bacteria [26], the OM-embedded portions of *TPA* OMPs adopt a β-barrel conformation in which extracellular loops (ECLs) bridge neighboring β-strands [23, 24, 27]. So-called ‘functional’ antibodies must target ECLs, the only antibody-accessible regions of OMPs, to promote clearance of spirochetes.

To study the antigenic properties of individual ECLs in a conformationally native-like state, they must be tethered, typically done using protein scaffolds [28–30]. We recently described the use of *Pyrococcus furiosus* thioredoxin (*Pf*Trx) as a scaffold for assessing the reactivity of *TPA* OMP ECLs with syphilitic sera and generating loop-specific, opsonic antibodies [18, 27]. We selected *Pf*Trx as a scaffold given its demonstrated success in generating neutralizing antibodies against epitopes in the L2 capsid protein of human papillomavirus, its versatility in accommodating loops of various sizes, its ease of expression and purification, and its exceptional stability [31, 32]. Herein, we used immune rabbit sera (IRS) to assess the immunogenicity of scaffolded ECLs from three newly discovered OMP paralogous families: OMFs, 8SβBs, and FadLs [23, 24, 27]. With this strategy, we identified five immunodominant ECLs from three members of the FadL family and used *Pf*Trx-scaffolded ECLs to generate opsonic antibodies in rabbits and mice. By exploiting the recent breakthrough in long-term *in vitro* cultivation of *TPA* [44], we discovered that rabbit and mouse opsonic antibodies against immunodominant FadL ECLs affected, to varying extents, spirochete viability, motility, and attachment to rabbit epithelial cells in the absence of active complement. Collectively, our findings support a strategy for syphilis vaccine development based upon targeting of ECLs, and they provide novel insights into the mechanisms whereby antibodies against *TPA* surface epitopes promote spirochete clearance.

## Results

### Prediction of ECL boundaries using structural models generated by trRosetta

**Fig 1:**
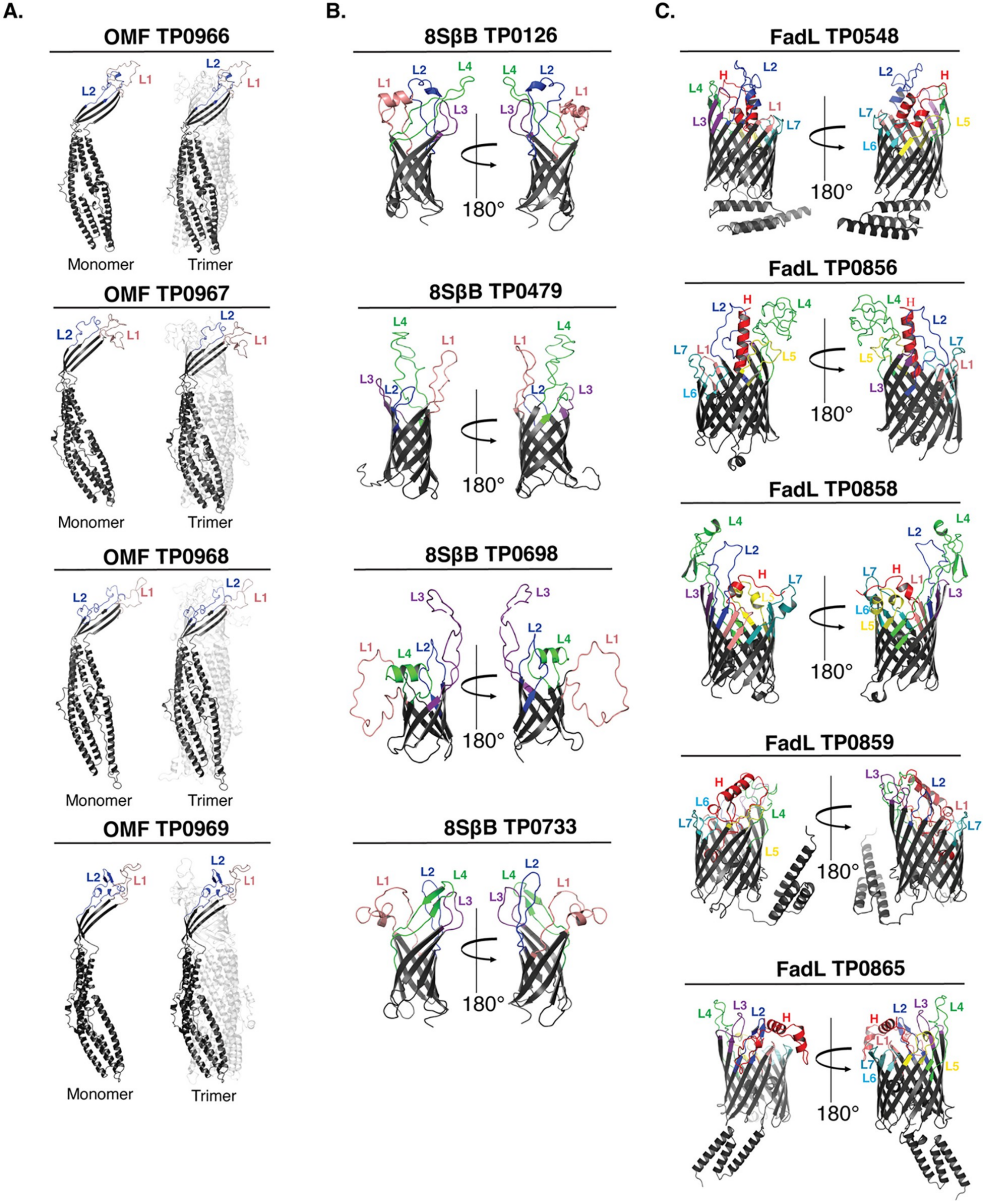
Prediction of ECL boundaries. trRosetta 3D models for outer membrane factors (OMFs), eight-stranded β-barrels (8SβBs), and FadLs (Panels A-C, respectively), depict ECL boundaries (ECL1-Salmon, ECL2-Blue, ECL3-Purple, ECL4-Green, ECL5-Yellow, ECL6-Cyan, ECL7-Dark Teal, and Hatch-Red) used to clone ECLs onto the *Pf*Trx scaffold (see **S1 Table**).

Previously, we used trRosetta [34] to generate three-dimensional (3D) structural models for three recently discovered *TPA* OMP paralogous families: OMFs, 8SβBs, and FadLs (**Fig 1**) [23, 24, 27]. These 3D models enabled us to identify the putative ECL boundaries (**S1 Table**) needed to create *Pf*Trx-scaffolded ECLs for the antigenicity analyses described below. Predictions by trRosetta and AlphaFold3 were consistent for the *TPA* 8SβB and FadL families; however, the *TPA* OMFs diverged substantially (**S1 Fig**). Structurally characterized OMFs are homotrimers in which the monomers contain four β-strands, two ECLs, and six extended α-helices (see examples *E*. *coli* TolC and *Neisseria gonorrhoeae* MtrE in **S1 Fig**) [35, 36]. trRosetta predicts canonical monomeric structures for all four *TPA* OMFs (TP0966, TP0967, TP0968, and TP0969). In contrast, AlphaFold3 models each monomer with eight β-strands, four small ECLs, and six α-helices. Based on the many solved structures available, we concluded that the trRosetta prediction of ECL boundaries are more likely to be correct and, therefore, used the four β-stranded monomer to complete the trimeric models using WinCoot [24] (**Fig 1A**).

### Antigenic analysis of scaffolded ECLs with *TPA* Nichols IRS reveals immunodominant FadL ECLs

**Fig 2:**
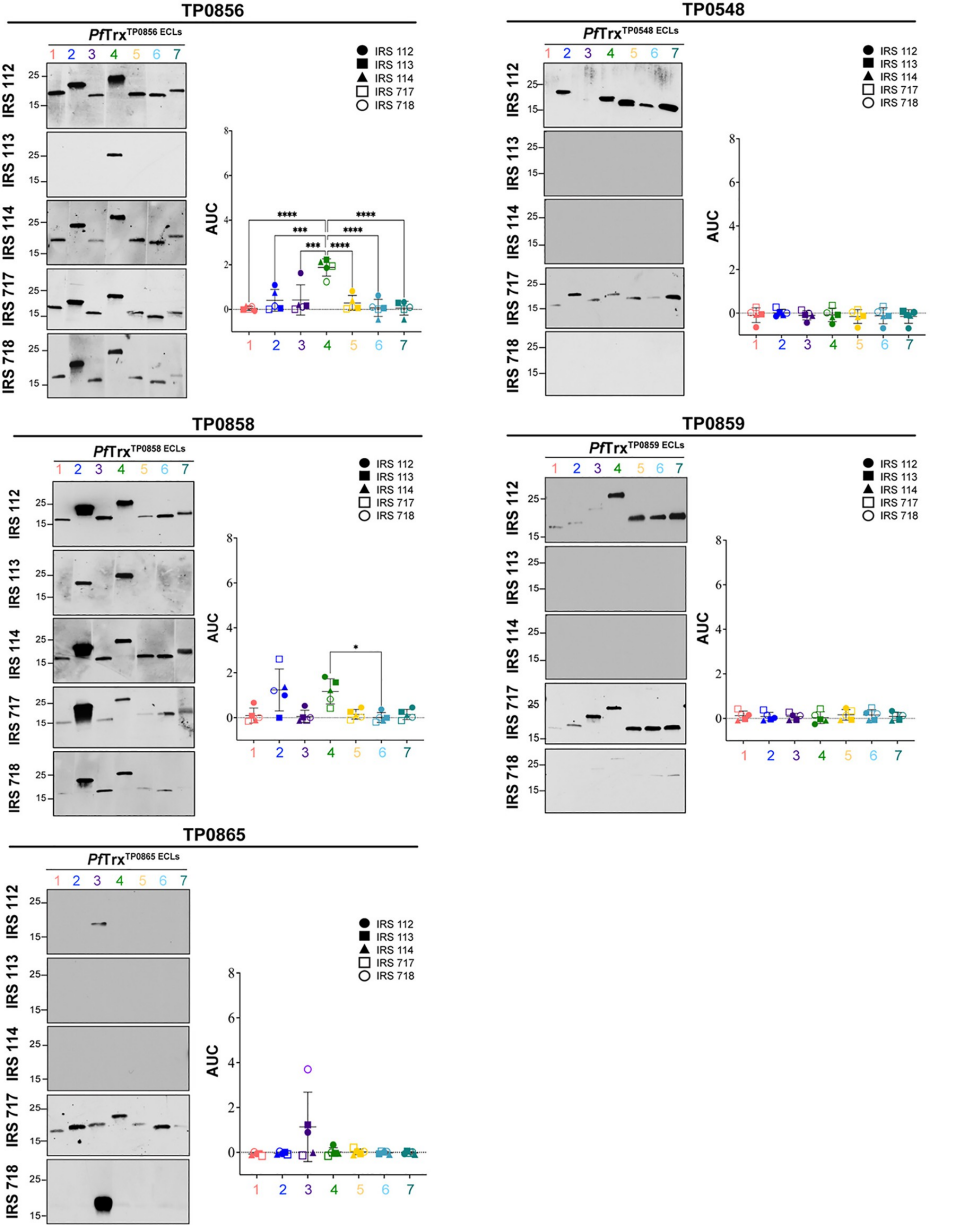
Reactivity of scaffolded ECLs with Nichols IRS reveals immunodominant FadL ECLs. Reactivity by immunoblot (left) and ELISA (right) of scaffolded FadL ECLs against sera from five Nichols immune rabbits. ELISA reactivity was measured as AUC corrected for *Pf*Trx background (see Methods). *n*  =  3 wells per condition. Data are shown as mean ± SD. Significant differences (*p<0.05; ***p<0.001; or ****p<0.0001) between the means of the groups were determined by one-way ANOVA with Bonferroni’s correction for multiple comparisons. Color codes of ECLs are as follows: ECL1-Salmon, ECL2-Blue, ECL3-Purple, ECL4-Green, ECL5-Yellow, ECL6-Cyan, and ECL7-Dark Teal.

Before proceeding to the examination of ECLs, we first assessed the reactivity of five Nichols IRS by immunoblotting against whole cell lysates from the same strain (**S2A Fig**). While each serum reacted strongly with known immunogenic lipoproteins (*e*.*g*., Tpp47, Tpp17, and Tpp15) [37, 38], we noted differences in their recognition of other *TPA* proteins as would be expected for outbred animals. We then examined the reactivity of scaffolded OMF, 8SβB, and FadL ECLs with the immune sera by immunoblot and ELISA. Not surprisingly, the five immune rabbits exhibited considerable heterogeneity in antibody responses to ECLs of all three OMP families. The OMF and 8SβB ECLs showed poor reactivity overall (**S3 Fig**). We also noted discordances between immunoblot and ELISA results for several OMF ECLs. For example, the strong ELISA reactivity of IRS 112 and 718 with both ECLs of TP0966 contrasted with their faint reactivity by immunoblot. Conversely, IRS 112 reacted strongly by immunoblot with ECL1 of TP0968 and ECL2 of TP0969 but showed no reactivity by ELISA (**S3A Fig**). Similar discordances were noted for the 8SβBs (**S3B Fig**). IRS 112 exhibited strong immunoblot reactivity for several 8SβBs ECLs that were non-reactive by ELISA, while IRS 113 reacted strongly by ELISA with ECL4 of TP0698 but showed no reactivity by immunoblot (**S3B Fig**). ECLs of the FadLs TP0856, TP0858, and TP0865 were the most immunoreactive overall (**Fig 2**). ECL2 and ECL4 of TP0856 and TP0858, along with ECL3 of TP0865, displayed strong reactivity by both immunoblot and ELISA.

**Fig 3:**
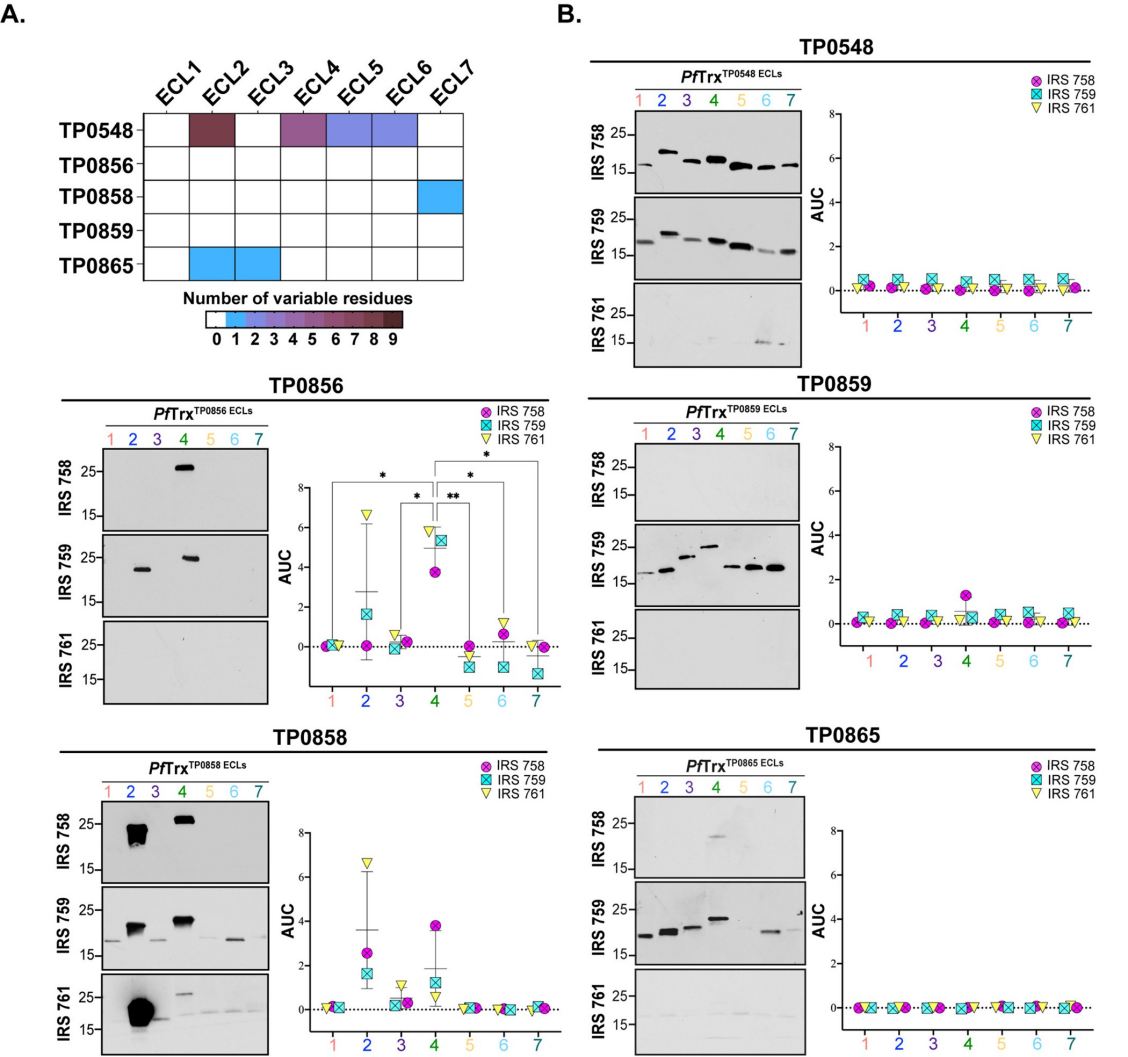
Comparative sequence analysis of the Nichols and SS14 FadLs and reactivity of Nichols FadL ECLs with SS14 IRS. (**A**) Summary chart representing the number of variable residues within Nichols and SS14 FadLs. (**B**) Reactivity by immunoblot (left) and ELISA (right) of Nichols FadL ECLs with SS14 IRS. ELISA reactivity measured as AUC corrected for *Pf*Trx background. *n*  =  3 wells per condition. Data are shown as mean ± SD. Significant differences (*p<0.05 or **p<0.01) between the means determined by one-way ANOVA with Bonferroni’s correction for multiple comparisons. Color codes of ECLs are as follows: ECL1-Salmon, ECL2-Blue, ECL3-Purple, ECL4-Green, ECL5-Yellow, ECL6-Cyan, and ECL7-Dark Teal.

Genomic sequencing has revealed that syphilis spirochetes cluster into two taxonomic groups represented by the Nichols and SS14 reference strains, with SS14-like strains predominating globally [39–41]. Given the epidemiologic importance of the SS14 clade, we next sought to determine whether SS14 immune rabbits also generate antibodies against FadL ECLs. We first assessed the reactivity of three SS14 immune sera by immunoblotting against Nichols *TPA* lysates (**S2A Fig**). As with the Nichols IRS, SS14 immune sera reacted strongly with known immunogenic lipoproteins, although, once again, minor differences were noted in their recognition of other *TPA* proteins. The antigenic profile of SS14 IRS with Nichols ECLs closely mirrored that observed with Nichols IRS, with ECL2 and ECL4 of TP0856 and TP0858 again the antigenic standouts (**Fig 3B**). TP0865 ECL3, on the other hand, demonstrated no ELISA reactivity with SS14 IRS. The observed difference in reactivity for TP0865 led to an evaluation of the amino acid sequence alignment between the Nichols and SS14 FadL orthologs (**S4 Fig**). A summary of the amino acid differences between the Nichols and SS14 strains for all FadL orthologs is presented in **Fig 3A**. This analysis revealed two notable changes in SS14 TP0865: a non-conservative substitution in ECL2 (A193T) and the insertion of an asparagine residue at position 238 in ECL3. In contrast, TP0856 and TP0859 are fully conserved at the amino acid level. TP0548, however, exhibited significant variability across four extracellular loops (ECLs 2, 4, 5, and 6). SS14 TP0858 contains a single conservative amino acid substitution in ECL7 (S380N) compared to the Nichols ortholog. Immunoblot and ELISA with an SS14 TP0865 ECL3 construct revealed that the lack of reactivity with Nichols TP0865 ECL3 was due to the absence of antibodies, not sequence variation (**S5 Fig**).

### FadL loop-specific antibodies from rabbits and mice exhibit opsonic activity

**Fig 4:**
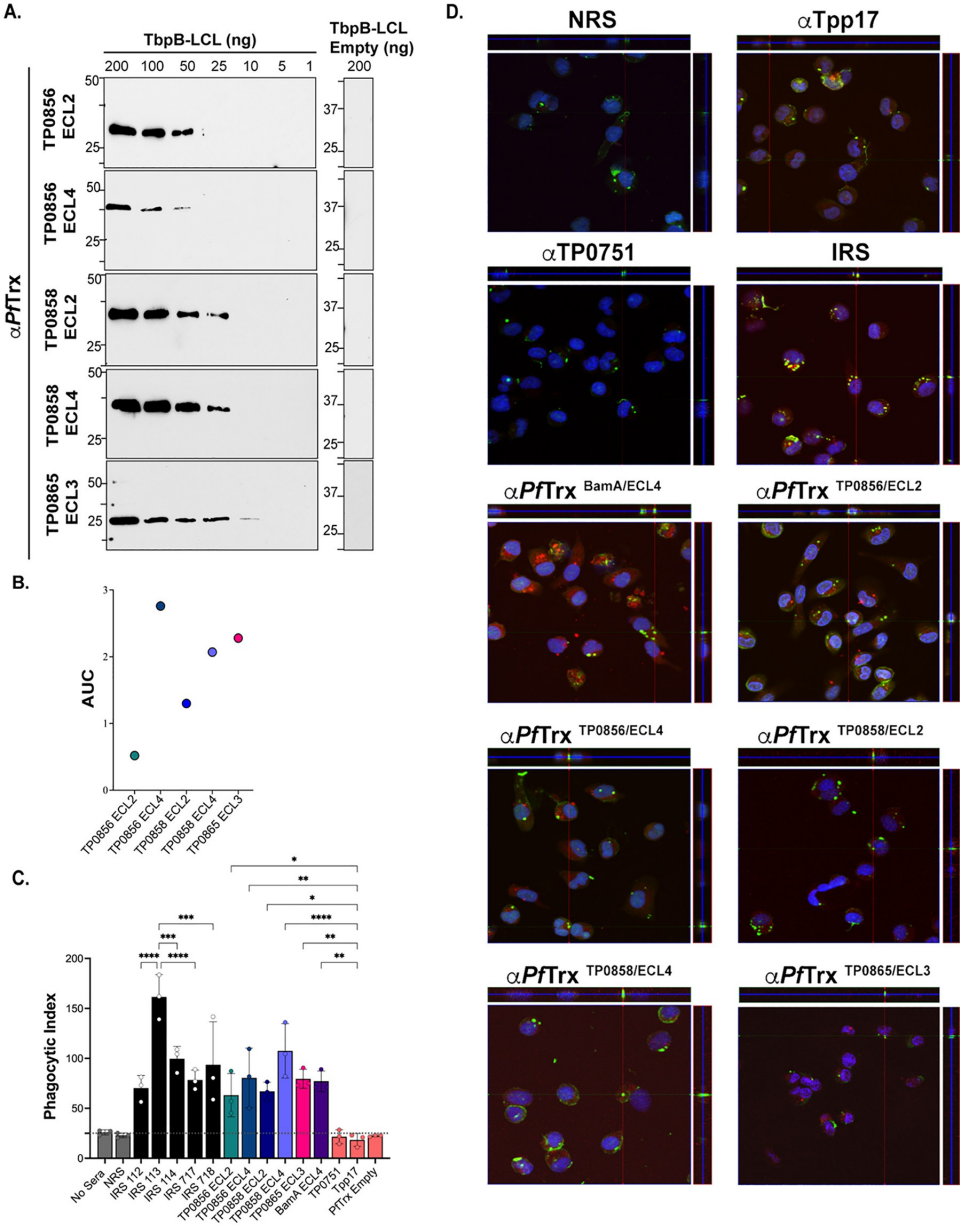
Opsonic activity of rabbit antisera to *Pf*Trx-scaffolded FadL ECLs. (**A**) Immunoblot and (**B**) ELISA (AUC) reactivities of rabbit ECL antisera against the corresponding Tbpb-LCL^ECL^ and Tbpb-LCL^Empty^. (**C**) *TPA* freshly harvested from rabbits was pre-incubated for 2 h with 10% heat-inactivated NRS, IRS, or rabbit antisera to *Pf*Trx ECLs, Tpp17, or TP0751 followed by incubation with rabbit peritoneal macrophages for 4 h at an MOI 10:1. Phagocytic indices were determined from epifluorescence micrographs as described in Methods [18]. Significant differences (*p<0.05, **p<0.01, ***p<0.001 or ****p<0.0001) were determined by one-way ANOVA using Newman-Keuls correction for multiple comparisons. Bars represent mean ± SD, *n* = 3 wells per condition. (**D**) Representative confocal micrographs showing composites of 9–12 consecutive Z-stack planes with labeling of *TPA*, plasma membranes, and nuclei shown in green, red and blue, respectively.

We next sought to determine whether immunization with *Pf*Trx scaffolds displaying immunodominant FadL ECLs would elicit antibodies that recognize their native counterparts on *TPA*. We first confirmed the presence of loop-specific antibodies in the rabbit *Pf*Trx^ECL^ antisera by immunoblot and ELISA against the corresponding loops displayed by a heterologous TbpB-LCL scaffold (**Fig 4A** and **4B**) [18, 29]. It was noteworthy that there did not appear to be a strict correlation between the two assays. For example, *Pf*Trx^TP0856/ECL4^ antibodies exhibited the strongest ELISA reactivity, yet showed the weakest reactivity by immunoblot. Conversely, *Pf*Trx^TP0856/ECL2^ antibodies displayed strong reactivity by immunoblot but the lowest reactivity by ELISA (**Fig 4A** and **4B**). The similar amino acid sequences of the ECL2s and ECL4s in TP0856 and TP0858 (**S6 Fig**) raised the possibility that each ECL might react with the corresponding heterologous antiserum. We eliminated this concern by using the TbpB-LCL scaffolded ECLs. The ECL2s displayed virtually no cross-reactivity by immunoblot and only weak cross-reactivity by ELISA (**S6A Fig**). Similarly, only weak cross-reactivity was observed for the ECL4s by both immunoblot and ELISA (**S6B Fig**).

For the rabbit opsonophagocytosis assays, heat-inactivated sera were used throughout to eliminate effects attributable to complement. Sera from the five Nichols immune rabbits and rabbit antiserum against *Pf*Trx-scaffolded ECL4 of BamA/TP0326 (*Pf*Trx^BamA/ECL4^), previously demonstrated to be strongly opsonic [18, 42], served as positive controls; normal rabbit sera (NRS), rabbit α-*Pf*Trx^Empty^, and α-Tpp17 and α-TP0751, previously shown to be non-opsonic [18, 43], were the negative controls. Internalization of spirochetes was assessed using confocal microscopy and quantified by calculating the phagocytic index as described previously [18] and in Methods. Compared to the negative controls, all five IRS exhibited significant opsonic activity, with IRS 113 displaying significantly greater opsonic activity relative to the other four. All five *Pf*Trx FadL ECL antisera demonstrated opsonic activity comparable to IRS; α-*Pf*Trx^TP0858/ECL4^ displayed the most robust opsonic activity (p<0.0001) relative to the negative controls (**Fig 4C** and **4D**).

**Fig 5:**
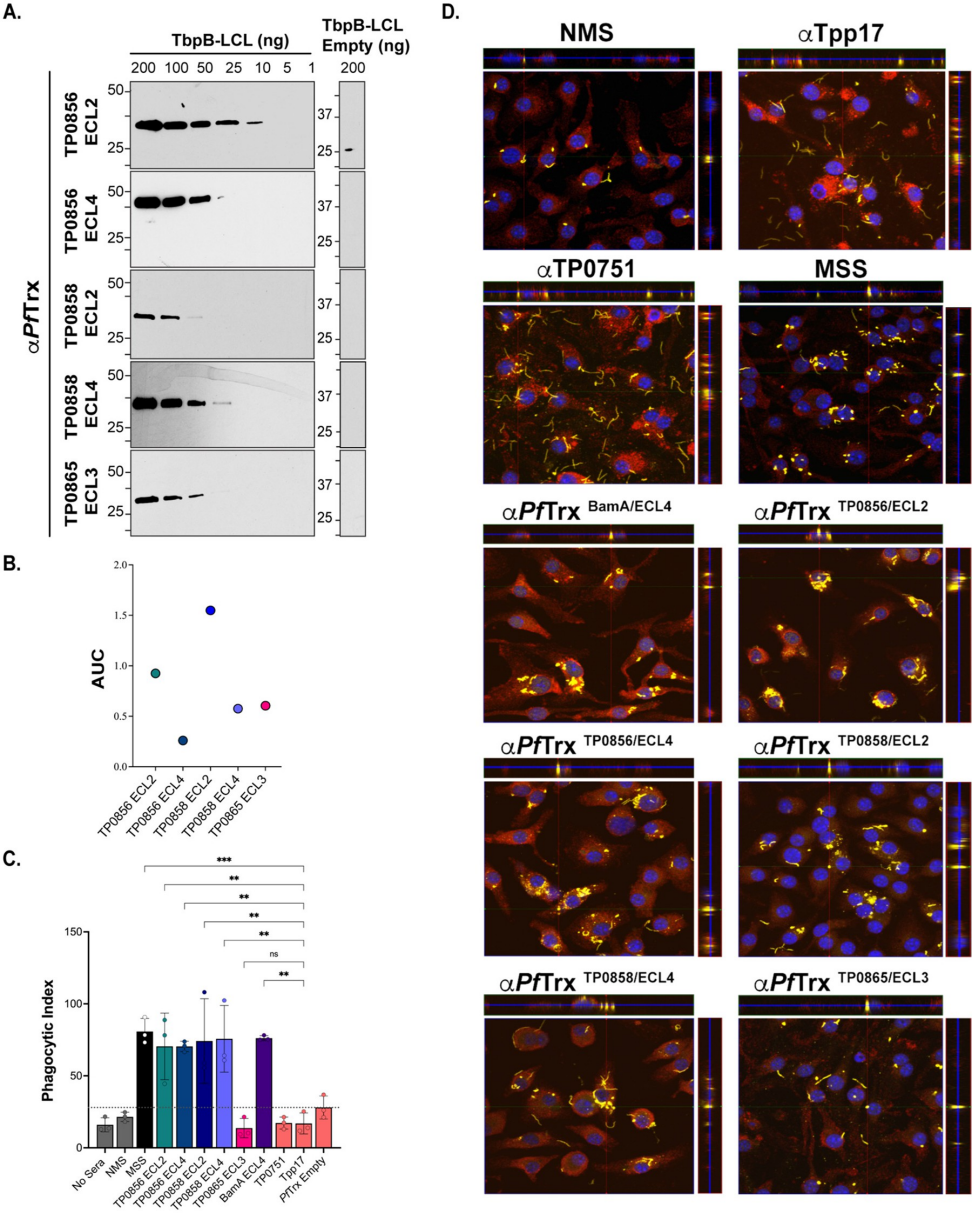
Opsonic activity of mouse antisera to *Pf*Trx-scaffolded FadL ECLs. (**A**) Immunoblot and (**B**) ELISA (AUC) reactivities of sera from mice immunized with *Pf*Trx scaffolded TP0856 ECL2 and ECL4, TP0858 ECL2 and ECL4, and TP0865 ECL3 against graded the corresponding Tbpb-LCL^ECL^ and Tbpb-LCL^Empty^. (**C**) *TPA* freshly harvested from rabbits were pre-incubated for 2 h with 10% heat-inactivated NMS, MSS, and mouse antisera against *Pf*Trx ECLs, Tpp17, and TP0751 followed by incubation with mouse BMDMs for 4 h at an MOI 10:1. Internalization of spirochetes was quantified from epifluorescence micrographs using the phagocytic index. Significant differences (*p<0.05 or **p<0.01) were determined by one-way ANOVA using Bonferroni’s correction for multiple comparisons. Bars represent mean ± SD, *n* = 3 wells per condition. (**D**) Representative confocal micrographs showing composites of 9–12 consecutive Z-stack planes with labeling of *TPA*, plasma membranes, and nuclei shown in yellow, red, and blue, respectively.

We recently described an opsonophagocytosis assay employing murine BMDMs to evaluate the opsonic activity of murine monoclonal and polyclonal ECL antibodies [18]. As before, we first confirmed the presence of loop-specific antibodies in pooled murine *Pf*Trx^ECL^ antisera by immunoblot and ELISA (**Fig 5A** and **5B**). Immunoblot analysis revealed that three of the five murine ECL antisera (α-*Pf*Trx^TP0856/ECL2^, α-*Pf*Trx^TP0856/ECL4^, and α-*Pf*Trx^TP0858/ECL4^) exhibited comparable sensitivity to their rabbit counterparts, while two (α-TP0858 ECL2 and α-TP0865 ECL3) displayed slightly lower reactivity (**Fig 5A**). As with the rabbit ECL antisera, immunoblot and ELISA results obtained with the two assays did not consistently correlate. For example, antibodies generated by *Pf*Trx^TP0856/ECL4^ exhibited the weakest ELISA reactivity, despite strong immunoblot reactivity, while *Pf*Trx^TP0858/ECL2^ displayed the strongest ELISA reactivity but low immunoblot reactivity (**Fig 5A** and **5B**). As with the rabbit assay, all sera were heat-inactivated to eliminate complement activity. Controls in the murine assay were analogous to the control rabbit sera (NMS, mouse α-*Pf*Trx^Empty^, and mouse α-Tpp17 and α-TP0751 as negative controls and mouse syphilitic sera (MSS), α-*Pf*Trx^BamA ECL4^ as positive controls). Four of the five pooled mouse *Pf*Trx ECL antisera (ECLs 2 and 4 of TP0856 and TP0858) exhibited significant opsonic activity comparable to the pooled MSS. Unlike its rabbit counterpart, mouse α-*Pf*Trx^TP0865/ECL3^ was not opsonic (**Fig 5C** and **5D**).

### Immune sera and loop-specific antibodies exhibit Fc receptor-independent functional activity against *in vitro* cultivated *TPA*

**Fig 6:**
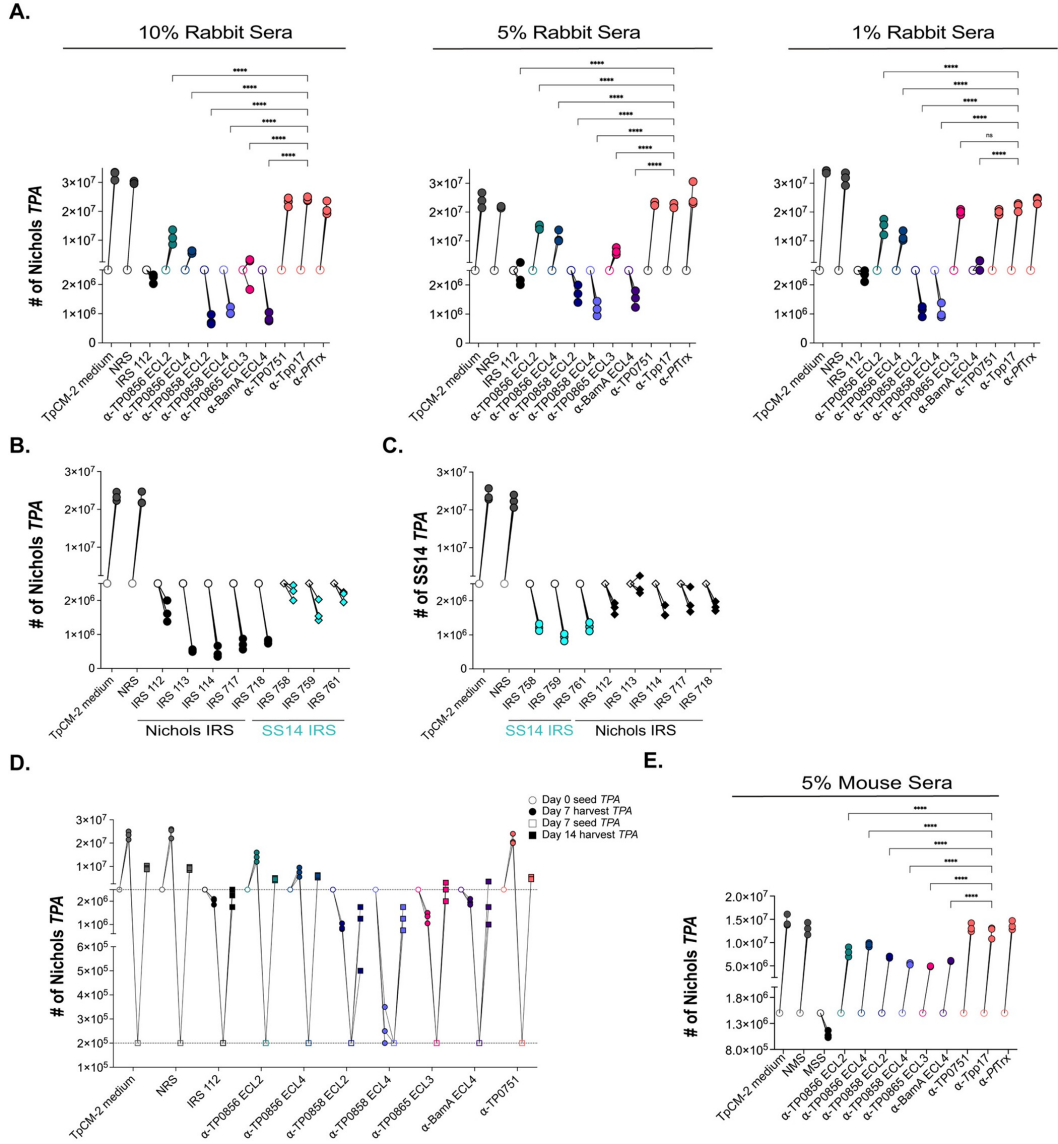
Rabbit and mouse antibodies impact growth of *TPA* Nichols and SS14 during *in vitro* cultivation. (**A**) Enumeration by darkfield microscopy (DFM) at day 7 (solid circles) of spirochetes cultured with 10%, 5%, and 1% concentrations of the indicated rabbit sera, with initial seeding at 2.5 x 10^6^ per well (open circles). (**B**) Nichols and (**C**) SS14 *TPA* strains were seeded initially at 2.5 x 10^6^ per well (open shapes) and cultured with Nichols and SS14 IRS (black and cyan, respectively). On day 7 (solid shapes), spirochetes were harvested and enumerated. Homologous IRS and heterologous IRS are depicted as circles and diamonds, respectively. (**D**) Spirochetes harvested on day 7 (solid circles) were transferred to a fresh plate containing Sf1Ep cells and TpCM-2 without rabbit sera. On day 14 (solid squares), spirochetes were harvested and enumerated. (**E**) Enumeration by DFM of spirochetes (initial seeding 1.5 x 10^6^ per well) with 5% mouse antisera targeting FadL ECLs. On day 7, samples were harvested for analysis as described above. Each condition was performed with n = 3 replicates. Significant differences (****p<0.0001) were determined by two-way ANOVA with Tukey correction for multiple comparisons.

As described in Methods, we modified the recently developed system for continuous *in vitro* propagation of *TPA* [33, 44] to investigate whether heat-inactivated IRS and loop-specific antibodies exert Fc receptor-independent functional activity against live *TPA*. Incubation of spirochetes with 10%, 5%, and 1% IRS 112 resulted in a reduction of spirochete numbers below the input level, accompanied by a striking loss of motility and showed severe deterioration of spirochetes (**Fig 6A** and **S1 Movie**), whereas NRS was without effect (**Fig 6A** and **S2 Movie**). Furthermore, unlike NRS, all incubations with IRS contained debris (**S1** and **S2 Movies**). This observation, coupled with the decreased number of spirochetes, points to a bactericidal activity of IRS resulting in spirochete lysis. In our hands, approximately 80% of spirochetes are adherent to the epithelial cells at the time of passage (**S2 Table**). At all three concentrations, IRS also markedly decreased attachment (~31% attached; *p*<0.0001). In accord with the opsonophagocytosis assays, we saw no effect on growth, motility, or attachment when spirochetes were cultured with α-Tpp17 or α-TP0751 (**Fig 6A**, **S3** and **S4 Movies,** and **S2 Table**). An important question is whether heterologous IRS exerts functional activity in this *in vitro* system. To address this, we compared the impact of incubation with homologous and heterologous heat-inactivated IRS on *in vitro* cultivated Nichols and SS14 *TPA*. Spirochete numbers fell below input levels and motility decreased following incubation of both reference strains with heterologous IRS although the effect was more pronounced with homologous IRS (**Fig 6B** and **6C**).

To examine the *in vitro* functional activity of graded concentrations of heat-inactivated loop-specific antibodies (**Fig 6A**), we began with rabbit antibodies against BamA ECL4, a known target of bactericidal antibodies in *E*. *coli* [45]. In contrast to α-*Pf*Trx^Empty^, incubation of spirochetes with α-*Pf*Trx^BamA/ECL4^ at 10% and 5% resulted in numbers below input levels, whereas growth was static following incubation with 1% α-*Pf*Trx^BamA/ECL4^. All three concentrations resulted in the presence of debris, loss of motility, and a substantial decrease in spirochete attachment (**Fig 6A**, **S2 Table**, and **S5 Movie**). At all three concentrations, incubation with α-*Pf*Trx^TP0858/ECL2^ and α-*Pf*Trx^TP0858/ECL4^ led to reduced spirochete numbers and loss of motility, whereas neither antiserum interfered with attachment. In contrast, only 10% α-*Pf*Trx^TP0865/ECL3^ affected spirochete numbers, motility, and attachment. Significantly, debris was consistently observed with spirochetes incubated with antisera against TP0858 ECLs at all concentrations and 10% α-*Pf*Trx^TP0865/ECL3^. Surprisingly, α-*Pf*Trx^TP0856/ECL2^ and α-*Pf*Trx^TP0856/ECL4^ only modestly affected spirochete growth and had no effect on motility, with only α-*Pf*Trx^TP0856/ECL2^ diminishing attachment (**Fig 6A** and **S2 Table**).

We next sought to determine whether spirochetes could recover from incubation with IRS and α-loop antibodies. In these experiments, we reduced the input organisms into wells without antibodies to 2 x 10^5^ spirochetes to compensate for the lower number of treponemes recovered from cultures with IRS and some ECL antibodies. While we observed recovery of spirochetes initially cultured with IRS and ECL antisera, none reached counts comparable to those of spirochetes initially incubated with NRS or *TPA* culture medium 2 (TpCM-2) (**Fig 6D**).

Lastly, we asked whether heat-inactivated opsonic murine antibodies are functional in the *in vitro* cultivation system. Due to the limited availability of mouse sera, the assay was scaled down and performed at a single concentration (*i*.*e*., 5%). At this concentration, MSS reduced total spirochete numbers below the initial seeding amounts and significantly impaired attachment (**Fig 6E and S3 Table**). As expected, NMS and mouse α-Tpp17 and α-TP0751 lacked activity (**Fig 6E**). Interestingly, unlike IRS, MSS did not significantly affect motility and did not result in the presence of debris (**S6–S9 Movies**). In contrast to the rabbit ECL antisera, none of the mouse ECL antisera, including α-*Pf*Trx^BamA/ECL4^, completely inhibited spirochete growth. Notably, the partial inhibition of growth seen with mouse α-*Pf*Trx^TP0856/ECL2^, α-*Pf*Trx^TP0856/ECL4^, and α-*Pf*Trx^TP0865/ECL3^ was comparable to that observed with the corresponding rabbit antisera (**Fig 6E**). Unexpectedly, none of the mouse ECL antisera, including α-*Pf*Trx^BamA/ECL4^ (**S10 Movie**), affected motility or resulted in debris, while all significantly affected attachment to varying degrees (**S3 Table**).

### Transcriptional analysis confirms expression of OMP targets *in vivo* and *in vitro*

**Fig 7:**
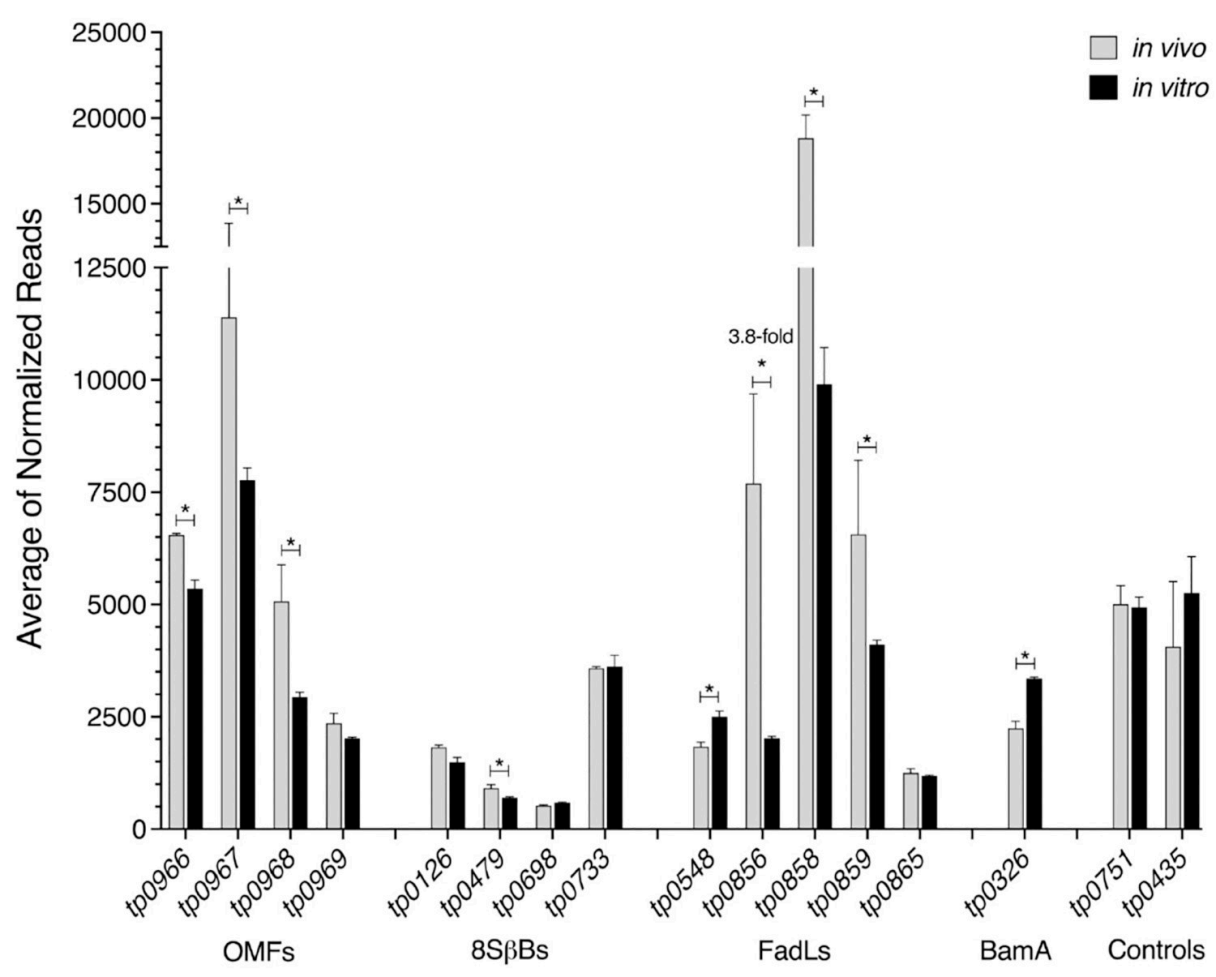
Transcriptional analysis of *TPA* OMP genes. Pairwise normalized read counts for OMP genes *in vivo* (grey) and *in vitro* (black) were calculated using DESeq2 after processing of raw reads previously published by De Lay *et al*. [46]. A *q*-value < 0.05, indicated with *, was considered as a significant difference between normalized counts *in vivo* compared to *in vitro*. Only one gene, *tp0856*, was expressed at ≥2-fold higher/lower levels in *TPA in vivo* compared to *in vitro* (fold change indicated above the bars).

Interpretation of the functional activity of ECL antibodies requires knowledge of the expression levels of the corresponding OMPs. We took advantage of the previously published RNAseq data from De Lay *et al*. [46] to compare OMP transcript levels in spirochetes harvested from rabbits and during *in vitro* cultivation. Reads were mapped to the *TPA* genome as previously described for RNA-seq analysis of *Borrelia burgdorferi* and *Leptospira interrogans* [47, 48] and pairwise differential gene expression was calculated using DESeq2 [49]. OMP-encoding genes expressed at ≥2-fold higher/lower levels *in vivo* compared to *in vitro* with a False-discovery rate (FDR)-adjusted-*p* value (*q*) <0.05 were considered differentially expressed. As shown in **Fig 7**, transcripts for all OMPs studied herein were detected under both conditions. Although several genes were differentially expressed with *q*<0.05, only one gene, *tp0856*, was expressed at greater than two-fold levels *in vivo* than *in vitro*. The FadL *tp0858* and the OMF *tp0967* were the OMPs with the highest levels overall, with an average of 18,825 and 11,394 normalized read counts *in vivo*, respectively. Interestingly, *tp0326*, whose corresponding protein (BamA) was strongly targeted by ECL4 antibodies in our opsonophagocytosis and *in vitro* assays, was expressed at relatively low levels *in vivo* and *in vitro*. Several other OMP genes were expressed at comparably low levels under both conditions, among them *tp0865* whose corresponding protein also was well targeted by loop-specific antibodies (**Figs 2** and **7**).

## Discussion

The alarming global resurgence of syphilis in the twenty first century [1–3] has created an urgent need for a vaccine with worldwide efficacy [4, 5]. A crucial first step for syphilis vaccine development is the identification of *TPA* surface antigens targeted by the functional antibodies in immune sera [10–13]. Our strategy for mining IRS for surface-directed antibodies was guided by our understanding of the molecular architecture of the *TPA* outer membrane and the structural biology of its repertoire of β-barrel forming OMPs [20–25]. We devised a ‘learning from nature’ variant of rational vaccine design in which the host’s natural immune response to a pathogen guides the selection of antigens [50, 51] by employing ECLs scaffolded by *Pf*Trx. This approach enabled us to sidestep cumbersome experimentation with full-length OMPs and focus instead on their antibody accessible regions. The success of the approach hinged on the structural models used to define ECL boundaries. Two lines of evidence supported the accuracy of the models. One was the agreement between trRosetta and AlphaFold3 for both 8SβBs and FadLs, alongside the similarity of predicted trRosetta OMFs to crystal structures of OMF orthologs of gram-negative bacteria. Secondly, binding of antibodies to the surface of motile treponemes, observed in two different assays with rabbit and mouse antisera, provided definitive evidence that the antigenic determinants presented by the scaffolds were extracellular.

ECLs can adopt stable conformations due to interactions with the barrel, with each other [52], or fixed structural elements within the loop [53], while others are mobile and flexible [54–56]. Structural characterization of loop-antibody complexes reveals that even mobile ECLs adopt specific conformations when bound by bactericidal antibodies [57]. Consistent with these prior studies, we discovered using IRS that tethered *TPA* ECLs possess a hitherto unsuspected degree of antigenic complexity. Instances where reactivity was observed by ELISA, but not by immunoblot (*e*.*g*., IRS 113 with TP0865 ECL3), revealed that IRS contains antibodies directed against discontinuous ECL epitopes and that the scaffolded loops can detect them. Conversely, ECLs that were detected by immunoblot, but not by ELISA (*e*.*g*., IRS 112 with TP0968 ECL1), likely indicate linear epitopes that are inaccessible or masked when ECLs are presented in a native-like state. Admittedly, we have not formally eliminated the possibility that the lack of ELISA reactivity reflects improper presentation of linear epitopes by the scaffolds. We consider this to be unlikely, however, given the ability of the loops to display discontinuous epitopes and the fact that antibodies generated with multiple scaffolded ECLs recognized their native counterparts on the *TPA* surface. Moreover, our previous work with BamA ECL4 revealed that the *Pf*Trx scaffolds can display linear epitopes in an antibody accessible manner [18]. During syphilitic infection, production of ECL antibodies that cannot ‘find’ their linear targets may be a novel manifestation of *TPA*’s capacity for antibody-evasiveness, a virulence trait we have designated ‘stealth pathogenicity’ [22]. While certain ECLs, particularly within the FadL family, exhibited robust antibody reactivity, others, most notably within the OMF and 8SβB families, exhibited poor antigenicity. Transcriptional data [46] suggest that these discordances may not be entirely attributed to differences in expression. A plausible explanation aligns with the presumed poor antigenicity of the syphilis spirochete’s rare OMPs–the central tenet of the stealth pathogenicity concept [23, 58–60].

Determination of an *in vitro* correlate of protection as an objective, quantitative criterion for a protective immune response is a prerequisite for the development of a vaccination strategy [61]. Strictly speaking, a true correlate of protection for syphilis does not yet exist. However, *in vivo* evidence from the rabbit model for macrophage-mediated clearance of *TPA* [10] has led to the widely accepted belief that antibodies that promote opsonophagocytosis of *TPA* can be considered a surrogate for a protective response [10, 14]. Studies conducted herein with sera from five immune rabbits demonstrated levels of *TPA* internalization greatly surpassing those observed with antibodies against the periplasmic controls, Tpp17 and TP0751 [38, 43]. It is interesting to note that the five immune sera from outbred rabbits exhibited a broad spectrum of reactivity to our panel of scaffolded ECLs. Collectively, these results point to the protective capacity of different combinations of loop antibodies. Furthermore, they suggest that examination of ECLs from additional members of the *TPA* OMPeome is warranted in the effort to create an optimally efficacious ECL vaccine cocktail.

Opsonophagocytosis of *TPA* is slow, inefficient, and incomplete [12, 62, 63]. These observations reflect not just the spirochete’s low density of OMPs but also their poor mobility [23, 64], a physical property that impedes the clustering required for Fc receptor signaling [65]. They also raise the question of whether the infected human host must deploy additional antibody-mediated functions to effect clearance of spirochetes. The recent breakthrough in long-term *in vitro* cultivation of *TPA* [33] provided a vehicle to assess whether surface-directed antibodies in IRS exert Fc receptor-independent activity against the syphilis spirochete. The detrimental impact of IRS on *TPA* growth and motility, together with the presence of debris not observed with NRS or periplasmic controls, suggested that IRS antibodies can exert bactericidal activity. Years ago, Nelson and Mayer [66] and Bishop and Miller [67] demonstrated *in vitro* complement-dependent killing of *TPA* by syphilitic sera. Azadegan *et al*. [68] showed that depletion of complement in hamsters accelerated lesion development following intradermal challenge and prevented protection following passive protection with immune hamster serum. Of note, heat-inactivation of all serum in this study supports that the observed Fc receptor-independent activities were also complement-independent. Nevertheless, the ability of spirochetes to recover once immune pressure was relieved points to the presence of a subpopulation of spirochetes capable of surviving the IRS antibody onslaught. This inference aligns with labeling experiments showing extreme variability in the degree of surface antibody binding by IRS within *TPA* populations [20] the survival of subpopulations of spirochetes during opsonophagocytosis experiments [12, 63], and passive-transfer experiments demonstrating the need for continuous administration of IRS to prevent lesion development [69, 70]. We also observed growth inhibition and lack of motility of Nichols and SS14 *TPA* cultured with homologous and heterologous IRS strains *in vitro*. These findings imply that antibodies directed against conserved ECL epitopes may result in cross-immunity. On the other hand, homologous IRS caused a more pronounced effect on *TPA* viability than heterologous IRS, supporting the importance of antibodies against variable surface epitopes for full protection. These results emphasize the importance of investigating multiple *TPA* strains, as the development of a broadly protective vaccine will likely require a combination of surface loops that can effectively target different strains. That antibodies in IRS exert Fc receptor-dependent and -independent activities clearly works to the advantage of the host. Organisms immobilized by IRS would be ‘sitting ducks’ for tissue macrophages.

The ‘learning from nature’ paradigm for vaccine design relies on the premise that immunization with immunogenic surface molecules identified in an immune serum will, if properly formulated, yield functional antibodies [50, 51]. This premise clearly was fulfilled for all five scaffolded, immunodominant FadL ECLs mined from IRS. Until recently [18], opsonophagocytosis assays were conducted with antibodies to full-length proteins or protein domains [42, 71–74]; positive results with these antigens left open the question of the precise surface location of the opsonic epitopes. Use of ECLs resolves this issue at a topological level though the specific residues involved in antibody binding still needs to be determined structurally. Opsonophagocytosis requires that antibodies bind to the bacterial surface for recognition by Fc receptors; the antibodies, however, are not the effectors. Studies with the *in vitro* cultivation system revealed that antibodies targeting specific ECLs can be true effectors, presumably interfering with the functions of individual OMPs to cause severe, even fatal, physiologic perturbations reflected by loss of motility and viability.

BamA is a central component of a molecular machine that cycles between open and closed states to insert newly synthesized OMPs into the OM bilayer [75]. ECL4 is part of a multi-loop dome that prevents egress of the OMP substrates to the external milieu [75]. Presumably, antibody binding to ECL4 prevents movements within the dome needed to accommodate cycling of the BamA β-barrel, inflicting a fatal lesion by impairing OM biogenesis [45]. Growth inhibition and killing by anti-FadL loop antibodies conceivably reflects interference with uptake of essential small molecules. We observed an intriguing dichotomy with antibodies to ECLs 2 and 4 of TP0856 and TP0858. Antibodies to the TP0858 ECLs had a dramatic effect on *TPA* growth and survival while the effects of antibodies to the corresponding loops of TP0856 were comparatively weak. Given the similar opsonophagocytosis results with these same antibodies, differences in antibody binding seem implausible; more likely is that TP0856 is physiologically redundant within the *in vitro* environment. Antibodies against two ECLs, ECL4 of BamA and ECL2 of TP0856, had a pronounced effect on cellular attachment, though in the context of markedly different effects on growth and motility. Given BamA’s critical role in OMP biogenesis [23, 24], it seems reasonable to conjecture that the anti-adhesive effect of the BamA ECL4 antibodies resulted from a broad disruption of the *TPA* surface. In contrast, TP0856 antibodies could have interfered with a specific loop-dependent adhesive function. This supposition is in line with examples of bacterial OMPs involved in maintaining cellular homeostasis whose ECLs also have a virulence-related function as adhesins [76, 77]. The *in vitro* cultivation system promises to be an important addition to the syphilologist’s toolkit for dissecting the cytadhesive properties of *TPA* OMPs—an area of investigation at the nexus of vaccine development and syphilis pathogenesis.

The rabbit has been the animal model of choice for basic syphilis research for decades [6–8]. Our studies deciphering ECL antibody responses in animals with proven immunity to intradermal inoculation and then improving upon them by artificial immunization further demonstrate the model’s utility. Nevertheless, the outbred nature of the rabbit, the skyrocketing costs for purchase and maintenance, and the limited commercial availability of rabbit-specific reagents impose serious constraints at a time of great urgency for identification, refinement, and validation of protective targets. Historically, the lack of skin lesion development, the large inoculum required for infection, and the delayed time course for spirochete clearance have discouraged use of the mouse model [15, 16, 19]. Moreover, whether mice develop protective immunity following *TPA* infection has not yet been established. While the immunobiology of syphilis in the mouse may be less than optimal for pathogenesis studies, the evidence in hand points to the mouse as the obvious animal model for expediting vaccine research. *TPA*-infected mice generate antibodies that strongly promote phagocytosis of spirochetes by BMDMs [18], as well as antibodies that inhibit *TPA* growth *in vitro*. From these results, we can surmise that *TPA-*infected mice, like rabbits, develop antibodies directed against ECLs and that comparison of the two responses could be highly informative. Overall, however, the murine responses following immunization with scaffolded ECLs were less robust than those of rabbits; this was particularly evident from the *in vitro* cultivation experiments. From one perspective, these differences are advantageous since they can be exploited to pinpoint ECL epitopes most important for protective antibodies. On the other hand, strategies to improve them clearly will need to be devised before the mouse can take its place as a reliable screening tool. Despite lingering questions and historical prejudices, the mouse model brings to syphilis vaccinology unparalleled benefits, including cost-effectiveness, access to a vast array of reagents, and a wealth of inbred strains with precisely defined genetic backgrounds and fully characterized immunologic phenotypes.

## Materials and Methods

### Ethics statement

Animal experimentation was conducted following the *Guide for the Care and Use of Laboratory Animals* (8th Edition) in accordance with protocols reviewed and approved by the UConn Health Institutional Animal Care and Use Committee (AP-200351-0124, AP-200362-0124, AP-201085-1226, and AP-201086-1226) under the auspices of Public Health Service assurance number A3471-01 (D16-00295).

### OMP Modeling

Three-dimensional models for the OMFs (TP0966, TP0967, TP0968, and TP0969), 8SβBs (TP0126, TP0479, TP0698, TP0733), and FadLs (TP0548, TP0856, TP0858, TP0859, and TP0865) were retrieved from pre-existing models generated from Hawley *et al*. [24]. For all three families, structural models and ECL boundaries were re-examined using AlphaFold3 [78] (https://golgi.sandbox.google.com/). High-confidence models from AlphaFold3 and trRosetta for the 8SβBs and FadL families demonstrated strong agreement of ECL boundaries.

### Propagation of *TPA* and generation of immune rabbit sera

The *TPA* Nichols and SS14 reference strains (SS14 was generously provided by Dr. Steven Norris, McGovern Medical School, University of Texas Health Science Center at Houston) were propagated by intratesticular inoculation of five and three adult male New Zealand White (NZW) rabbits respectively as previously described [13, 20]. Immune rabbits were generated by inoculation of rapid plasma reagin-nonreactive adult NZW rabbits in each testis with 1 x 10^7^ treponemes in 500 μL CMRL containing 10% NRS. The immune status of each rabbit was confirmed sixty days post-inoculation by intradermal challenge with 1 x 10^3^ freshly extracted *TPA* (Nichols or SS14) at each of eight sites on their shaved backs. Immune sera were collected at monthly intervals thereafter.

### Generation of mouse syphilitic sera

Five male and five female six- to eight-week-old C3H/HeJ mice were inoculated intradermally, intraperitoneally, intrarectally, and intra-genitally with a total of 1 x 10^8^ total organisms per animal as previously described [17, 18]. Mice were sacrificed on day 84 post-inoculation and exsanguinated to create a pool of MSS.

### Cloning ECLs into *Pf*Trx and TbpB-LCL scaffolds

A codon-optimized version of *Pyrococcus furiosus* thioredoxin (*Pf*Trx) [27] with *TPA* BamA ECL4 inserted between amino acid residues 26 and 27 of the native *Pf*Trx and a C-terminal Avi-Tag (GLNDIFEAQKIEWHE) was synthesized by Genewiz. The resulting construct (*Pf*Trx^BamA/ECL4^) was PCR-amplified and cloned into NdeI-XhoI digested pET28a by In-Fusion cloning. To generate *Pf*Trx^Empty^, *Pf*Trx^BamA/ECL4^ was digested with BamHI to remove the ECL4-encoding DNA and then self-ligated. S1 Table contains the primers and sequences used to generate amplicons encoding ECLs other than BamA ECL4 for display by *Pf*Trx scaffolds (see below). *Pf*Trx scaffolds displaying ECLs shorter than 30 amino acids were generated by inverse PCR of pET28a^*Pf*Trx^ using primers containing the corresponding ECL sequences followed by InFusion cloning. *Pf*Trx constructs containing ECLs longer than 30 amino acids were generated by PCR-amplifying the loops from codon-optimized synthetic genes followed by insertion into BamHI-digested pET28a^*Pf*Trx^ by InFusion cloning. *Pf*Trx ECLs used for antigenic analyses (see below) were biotinylated during expression in *E*. *coli* BL21 (DE3) transformed with BirA (BPS Bioscience, San Diego, CA) [27].

DNAs encoding transferrin-binding protein B loopless C-lobe scaffold (TbpB-LCL) derived from *Neisseria meningitidis* TbpB [18, 29] and TbpB-LCL displaying *TPA* ECLs (**S1 Table**) were synthesized by Azenta Life Sciences (Burlington, MA) and cloned into pRB1B by In-fusion cloning as previously described [18, 27]. Plasmid inserts were confirmed by Sanger sequencing and then transformed into *E*. *coli* BL21-Gold (DE3) (Agilent, Santa Clara, CA) for overexpression. All constructs were purified over Ni-NTA resin (Qiagen, Germantown, MD) followed by size exclusion chromatography as previously described [27].

### Immunoblot analysis

#### IRS with *TPA* lysates

Nichols *TPA* lysates (5 x 10^7^ spirochetes per lane) were resolved by SDS-PAGE using a 4–20% gradient Any kD Mini-Protean TGX gels (Bio-Rad) and transferred to 0.45 nm nitrocellulose membranes (Bio-Rad, Hercules, CA). The membranes were blocked for 1 h with PBS containing 5% nonfat dry milk and 0.1% Tween 20 and probed overnight (ON) at 4°C with either Nichols or SS14 immune rabbit serum (IRS) (both at 1:1,000 dilutions) from individual rabbits. After washing with PBS containing 0.05% Tween 20 (PBST), the membranes were incubated for 1h at RT with HRP-conjugated goat anti-rabbit IgG or (1:30,000). Following further washes with PBST, the immunoblots were developed on a single film using the SuperSignal West Pico chemiluminescent substrate (ThermoFisher Scientific, Inc., Waltham, MA).

#### Reactivity of IRS and MSS with *Pf*Trx-scaffolded ECLs

400 ng of *Pf*Trx^Empty^, *Pf*Trx-scaffolded ECLs, and 20 ng of Tpp17 were incubated with 1:250 dilutions of Nichols or SS14 IRS or pooled Nichols MSS followed by HRP-conjugated goat anti-rabbit IgG or goat anti-mouse Ig (1:30,000) as described above.

#### Loop-specific antibodies in *Pf*Trx ECL antisera

Loop-specific reactivity of rabbit and mouse *Pf*Trx-ECL antisera was determined using TbpB-LCL from *Neisseria meningitis* as a second ECL scaffold as previously described [18]. Graded amounts of the corresponding TbpB-LCL-ECL (200 to 1 ng) were resolved as described above, and probed ON at 4°C with 1:1000 dilutions of rabbit or mouse *Pf*Trx-ECL antisera. After washing with PBST, the membranes were incubated for 1 h at RT with HRP-conjugated goat anti-rabbit IgG or goat anti-mouse Ig (1:30,000) as previously described [18]. 200 ng of TbpB-LCL^Empty^ was used as a negative control.

### ELISA analysis

#### Reactivity of IRS and MSS with *Pf*Trx-scaffolded ECLs

Clear Flat-Bottom Immuno Nonsterile 96-well plates (ThermoFisher Scientific, Inc.) were coated with streptavidin (SP; ThermoFisher Scientific, Inc.) diluted in 0.1M sodium bicarbonate (pH 8.5) at 200 ng/well and incubated ON at 4°C. After washing with 0.1% PBST, plates were blocked in PBS buffer containing 15% goat serum, 0.5% Tween 20, and 0.05% sodium azide (blocking buffer) for 1 h at RT. Biotinylated ECL scaffolded proteins were added at 200 ng/well in blocking buffer followed by incubation for 1 h at RT. After washing, either Nichols IRS, SS14 IRS, or MSS was added in 2-fold serial dilutions (1:20 starting dilution) in PBS with 1% bovine serum albumin (BSA) for 1 h incubation at RT. HRP-conjugated goat anti-rabbit IgG or goat anti-mouse Ig (1:10,000) then was added, followed by incubation for 1 h at RT. Plates were washed and developed with TMB single solution (ThermoFisher Scientific, Inc.). Reactions were stopped with 0.3M HCl. Area under the curve (AUC) for each scaffolded ECL were calculated following subtraction of the AUC for *Pf*Trx^Empty^.

#### Reactivity of rabbit ECL antisera with TbpB-LCL-scaffolded ECLs

Clear Flat-Bottom Immuno Nonsterile 96-well plates (ThermoFisher Scientific, Inc.) were coated with 6x-His tag monoclonal antibody (HIS.H8) (ThermoFisher Scientific, Inc.). All subsequent steps were performed as described above using 200 ng/well of TbpB-LCL-scaffolded ECL and a 2-fold serial dilution of *Pf*Trx^ECL^ antisera. The AUC for each scaffolded ECL was calculated following subtraction of the AUC for TbpB-LCL^Empty^.

#### Reactivity of mouse ECL antisera with TbpB-LCL-scaffolded ECLs

Mouse *Pf*Trx^ECL^ antisera was absorbed against TbpB-LCL^Empty^ using Dynabeads (CAT# 10103D, 10104D) according to the His-Tag Isolation & Pulldown protocol from Invitrogen. Clear Flat-Bottom Immuno Nonsterile 96-well plates (ThermoFisher Scientific, Inc.) were coated with 200ng/well of TbpB-LCL-scaffolded ECLS in PBS. Loop-specific antibodies were then assessed as described above in the rabbit antisera.

### Sequence alignment of Nichols and SS14 FadLs

Protein sequences for full length FadL orthologs or selected ECLs from the *TPA* Nichols (CP004010.2) and SS14 (CP004011.1) reference genomes were aligned using Clustal Omega [79].

### Immunization of rabbits and mice with *Pf*Trx-ECLs

For each ECL construct two adult male NZW rabbits were primed with a total of 200 μg of *Pf*Trx-scaffolded ECL in 500 μl of PBS-TiterMax (1:1, vol/vol) administered as four subcutaneous injections and two intramuscular injections with 100 μL and 50 μL, respectively. Rabbits were boosted at 3, 6, and 9 weeks with the same volumes and amounts of protein in PBS/TiterMax (1:1, vol/vol) and exsanguinated 12 weeks post-immunization. A total of 10 six- to eight-week-old C3H/HeJ mice (5 males and 5 females; Jackson Laboratory) were primed by intradermal injections with 100μl Freund’s Complete Adjuvant (1:1, v/v) containing 20 μg of ECL scaffolded proteins described above. Mice were boosted at 3, 5, and 7 weeks with the same volumes and amounts of protein in Freund’s Incomplete Adjuvant (1:1, v/v) and exsanguinated 9 weeks post-immunization. Sera from rabbits and pooled sera from mice were heat-inactivated, and then used in immunologic assays.

### Opsonophagocytosis assays

#### Generation of macrophages

Rabbit peritoneal macrophages were generated using 10% protease peptone and isolated using ice-cold PBS EDTA as previously described [18, 43]. The macrophages were plated at a final concentration of 1 x 10^**5**^ cells/well in 8-well BioCoat Poly-D-Lysine glass culture chamber slides (Corning, Corning, NY) and incubated at 37°C for 2 h. Nonadherent cells were removed by washing the monolayers twice with Dulbecco’s Modified Eagle Medium (DMEM) prior to the addition of *TPA*. Murine C3H/HeJ bone-marrow-derived macrophages (BMDM) were generated as previously described [17, 18], plated at a final concentration of 1 x 10^**5**^ cells per well in Millicell EZ 8-well chamber slides (Sigma-Aldrich, St. Louis, MO), and incubated ON at 37°C. The following day, the medium was replaced with fresh DMEM supplemented with 10% FBS prior to the addition of *TPA*.

**Opsonophagocytosis**. Freshly harvested *TPA* were diluted to 1 x 10^8^ per ml in DMEM or DMEM supplemented with 1:10 dilutions of heat-inactivated normal mouse or rabbit serum, mouse or rabbit syphilitic sera, or mouse or rabbit antisera directed against *Pf*Trx^TP0856/ECL2^, *Pf*Trx ^TP0856/ECL4^, *Pf*Trx ^TP0858/ECL2^, *Pf*Trx ^TP0858/ECL4^, *Pf*Trx ^TP0865/ECL3^, *Pf*Trx^Empty^. Negative controls included heat-inactivated rabbit and mouse α-Tpp17 and α-TP0751 sera [38,43]. Each condition was performed in triplicate. *TPA* was pre-incubated at RT for 2 h without or with sera followed by incubation for 4 h at 37°C with macrophages (plated as described above) at MOIs of 10:1.

#### Determination of spirochete uptake

Supernatants were removed, and rabbit peritoneal macrophages were fixed and permeabilized with 2% paraformaldehyde and 0.01% Triton X-100 for 10 mins at RT. Each well was rinsed with PBS and blocked with CMRL 10% normal goat sera (NGS) for 1 h at RT, and then incubated with MSS generated above (1:25) in CMRL 10% NGS ON at 4°C. After four successive washes with PBST, cells were blocked with CMRL 10% NGS for 1 h at RT, then incubated with α-mouse IgG AF488 (1:500) for 1 h at RT, followed by Cholera Toxin AF647 (1:500) for 30 min and DAPI (1:1000) for 10 min. After staining for *TPA*, the cells then were washed thoroughly three times with PBST, rinsed with deionized (DI) water to remove salt, and allowed to air dry. Finally, Vectashield (Vector Laboratories, Inc., Newark, CA) was added, and samples were sealed with a coverslip. Internalization of *TPA* was assessed in a blinded fashion by acquiring images of at least 100 macrophages per well on an epifluorescence Olympus BX-41 microscope [18]; images were processed with VisiView (version 5.0.0.7; Visitron Systems GmbH, Puchheim, Germany). The phagocytic index was calculated by dividing the number of internalized spirochetes by the total number of cells imaged and multiplying by 100 [18]. Confocal images were acquired using Zeiss 880 and processed using ZEN3.5 Blue. For IFA of murine BMDMs, cells were blocked with 5% BSA in PBS for 1 h at RT and then incubated with a commercially available rabbit α-*TPA* (1:100), ON at 4°C. the next day the cells were washed four times with PBST and incubated with a-rabbit IgG Texas Red (1:500) for 1 h at RT, followed by Phalloidin AF488 (1:10), Cholera Toxin AF647 (1:500) for 30 min, and DAPI (1:1000) for 10 min. Internalization of *TPA* was assessed as described above.

### Assessment of functional activity using *in vitro* cultivated *TPA*

Cottontail rabbit epithelial cells (Sf1Ep) [33, 44], generously provided by Drs. Diane Edmondson and Steven Norris (UT Health Science Center at Houston), were seeded at 2 x 10^4^ cells/well in a 24-well culture plate and incubated ON at 37°C. The following day, wells were washed once with *TPA* culture medium 2 (TpCM-2) [33, 44] equilibrated under microaerobic conditions (1.5% O_2_, 3.5% CO_2_, and 95% N_2_) followed by the addition of 2.5 ml of fresh TpCM-2 for a minimum of 3 h under microaerobic conditions. 2.5 x 10^6^ freshly harvested *TPA* were added to each well along with heat-inactivated normal sera, or syphilitic sera, or *Pf*Trx ECL antisera (*Pf*Trx ^TP0856/ECL2^, *Pf*Trx ^TP0856/ECL4^, *Pf*Trx ^TP0858/ECL2^, *Pf*Trx ^TP0858/ECL4^, *Pf*Trx ^TP0865/ECL3^). Control antisera included heat-inactivated *Pf*Trx^BamA/ECL4^, *Pf*Trx^Empty^, Tpp17 and TP0751. Spirochetes were harvested following incubation for seven days under microaerobic conditions. Supernatants were collected and set aside for subsequent DFM enumeration. Wells were then washed once with 200 μl of trypsin EDTA to remove traces of TpCM-2 media. 200 μl of Trypsin EDTA then was added to each well and incubated at 37°C for 5 min following which *TPA* released from the cells was collected in separate 5 ml conical tubes. The supernatant and cell-associated (*i*.*e*., trypsinized) fractions were centrifuged at 130 x *g* for 5 min followed by DFM enumeration. Movies following incubations were obtained using OCULAR Advanced Scientific Camera Control version 2.0 (64 bit) software with PVCAM version 3.8.0 (Teledyne Photometrics, Tucson, AZ)

To evaluate the viability of spirochetes following incubation with IRS and loop-specific antibodies, 2 x 10^5^ spirochetes per well were passaged to fresh wells containing Sf1Ep cells and fresh TpCM-2 medium for an additional 7 days followed by DFM enumeration. In these experiments, the number of input organisms was reduced to normalize for the lower numbers of treponemes harvested from day 7 cultures containing antibodies.

### Comparison of *in vivo* and *in vitro TPA* OMPs transcripts

Previously published [46] raw read sequencing data for *TPA* strain Nichols cultivated *in vitro* and harvested from infected rabbits were downloaded from the NCBI Sequence Read Archive (SRA) database (accession numbers SRR16297052, SRR16297053, SRR16297054, SRR16297055, SRR16297056, SRR16297057, SRR16297058 and SRR16297059). Reads were trimmed using Sickle version 1.3.3 (available from https://github.com/najoshi/sickle) [80] and then mapped using EDGE-pro version 1.1.3 [81] using FASTA, protein translation table (ptt) and ribosomal/transfer RNA table (rnt) files based on the *TPA* strain Nichols genome (RefSeq: NC_021490.2). Normalized read counts for each gene and differential gene expression were determined using DESeq2 [49].

### Statistical analysis

General statistical analysis was conducted using GraphPad Prism 9.5.1 (GraphPad Software, San Diego, CA). The means of the AUC from ELISA dilution curves for the *Pf*Trx-scaffolded ECLs constructs were compared by one-way ANOVA with Bonferroni’s correction for multiple comparisons. One-way ANOVA was used to compare phagocytic indices in rabbits and mice using Newman-Keuls and Bonferroni’s correction for multiple comparisons, respectively. A two-way ANOVA was used to compare *TPA* growth *in vitro* with Tukey correction for multiple comparisons in rabbits and mice assays. Ordinary one-way ANOVA was used to compare attached *TPA* in rabbits and in mice using Bonferroni’s correction for multiple comparisons. In gene expression studies, genes at ≥2-fold higher/lower levels in *TPA in vivo* compared to *in vitro* with a False Discovery Rate (FDR)-adjusted p-value (q-value) < 0.05 were considered differentially expressed. For each experiment, the standard error of the mean was calculated with *p*-values <0.05 considered significant.

## Supporting information

S1 Fig Comparison of OMF crystal structures with predicted models for TP0967 generated by AlphaFold3 and trRosetta. OMF crystal structures of *Neisseria gonorrhoeae* MtrE and *E*. *coli* TolC compared to trRosetta and AlphaFold3 three-dimensional models of OMF TP0967.
(PNG)

S2 Fig Immunoblot reactivity of Nichols and SS14 IRS with *TPA* Nichols lysates, *Pf*Trx^Empty^, and Tpp17. (**A**) Immunoblot reactivity of Nichols and SS14 IRS with Nichols lysates. (**B**) Immunoblot reactivity of Nichols and SS14 IRS with *Pf*Trx^Empty^ and Tpp17 proteins.
(PNG)

S3 Fig Reactivity of scaffolded ECLs with Nichols IRS reveals poorly antigenic OMF and 8SβB ECLs. Reactivity by immunoblot (left) and ELISA (right) of scaffolded ECLs of (**A**) OMFs and (**B**) 8SβBs against sera from five Nichols immune rabbits. ELISA reactivity was measured as area under the curve (AUC) corrected for *Pf*Trx background (see Methods). *n*  =  3 wells per condition. Data are shown as mean ± SD. Color codes of ECLs are as follows: ECL1-Salmon, ECL2-Blue, ECL3-Purple, ECL4-Green, ECL5-Yellow, ECL6-Cyan, and ECL7-Dark Teal.
(PNG)

S4 Fig Sequence alignments for FadL orthologs in *TPA* Nichols and SS14 reference strains. Clustal Omega alignments of the five Nichols FadL orthologs with highlighted variations shown in magenta. Predicted ECLs are indicated using color scheme described in **Fig 1**. Discontinuous BCE predictions by DiscoTope 2.0 and ElliPro are shown in purple boxes along the sequences.
(PDF)

S5 Fig Antigenic characterization of SS14 TP0865 ECL3. (**A**) Immunoblot and ELISA (AUC) reactivity of SS14 TP0865 ECL3 with (**B**) Nichols and (**C**) SS14 IRS.
(PNG)

S6 Fig Limited cross-reactivity of ECLs 2 and 4 of TP0856 and TP0858. (**A**) Alignment of TP0856 and TP0858 ECL2 sequences. Immunoblot and ELISA (bottom left and right, respectively) reactivity of rabbit anti-*Pf*Trx^TP0856/ECL2^ and anti-*Pf*Trx^TP0858/ECL2^ against TbpB-LCL^TP0856/ECL2^ and TbpB-LCL^TP0858/ECL2^. (**B**) Alignment of TP0856 and TP0858 ECL4 sequences. Immunoblot and ELISA (bottom left and right, respectively) of rabbit anti-*Pf*Trx^TP0856/ECL4^ and anti-*Pf*Trx^TP0858/ECL4^ against TbpB-LCL^TP0856/ECL4^ and TbpB-LCL^TP0858/ECL4^.
(PNG)

S1 Table ECL sequences and primer pairs used to generate *Pf*Trx-scaffolded ECLs. (PDF)

S2 Table Attachment of *in vitro* cultivated *TPA* Nichols following incubation for seven days with IRS and rabbit antisera. (PDF)

S3 Table Attachment of *in vitro* cultivated *TPA* Nichols following incubation for seven days with pooled MSS and mouse antisera. (PDF)

S1 Movie DFM of *in vitro* cultivated *TPA* Nichols incubated for seven days with 10% IRS. (MP4)

S2 Movie DFM of *in vitro* cultivated *TPA* Nichols incubated for seven days with 10% NRS. (MP4)

S3 Movie DFM of *in vitro* cultivated *TPA* Nichols incubated for seven days with 10% rabbit α-Tpp17. (MP4)

S4 Movie DFM of *in vitro* cultivated *TPA* Nichols incubated for seven days with 10% rabbit α-TP0751. (MP4)

S5 Movie DFM of *in vitro* cultivated *TPA* Nichols incubated for seven days with 10% rabbit α-*Pf*Trx^BamA/ECL4^. (MP4)

S6 Movie DFM of *in vitro* cultivated *TPA* Nichols incubated for seven days with 5% pooled MSS. (MP4)

S7 Movie DFM of *in vitro* cultivated *TPA* incubated for seven days with 5% pooled NMS. (MP4)

S8 Movie DFM of *in vitro* cultivated *TPA* Nichols incubated for seven days with 5% pooled mouse α-Tpp17. (MP4)

S9 Movie DFM of *in vitro* cultivated *TPA* Nichols incubated for seven days with 5% pooled mouse α-TP0751. (MP4)

S10 Movie DFM of *in vitro* cultivated *TPA* Nichols incubated for seven days with 5% pooled mouse α-*Pf*Trx^BamA/ECL4^. (MP4)
