# Supplementary figures and images for: Immunodominant extracellular loops of Treponema pallidum FadL outer membrane proteins elicit antibodies with opsonic and growth-inhibitory activities
Source: PLoS Pathog. 2024 Dec 23;20(12):e1012443. doi: 10.1371/journal.ppat.1012443 (PMC11761103; doi:10.1371/journal.ppat.1012443)

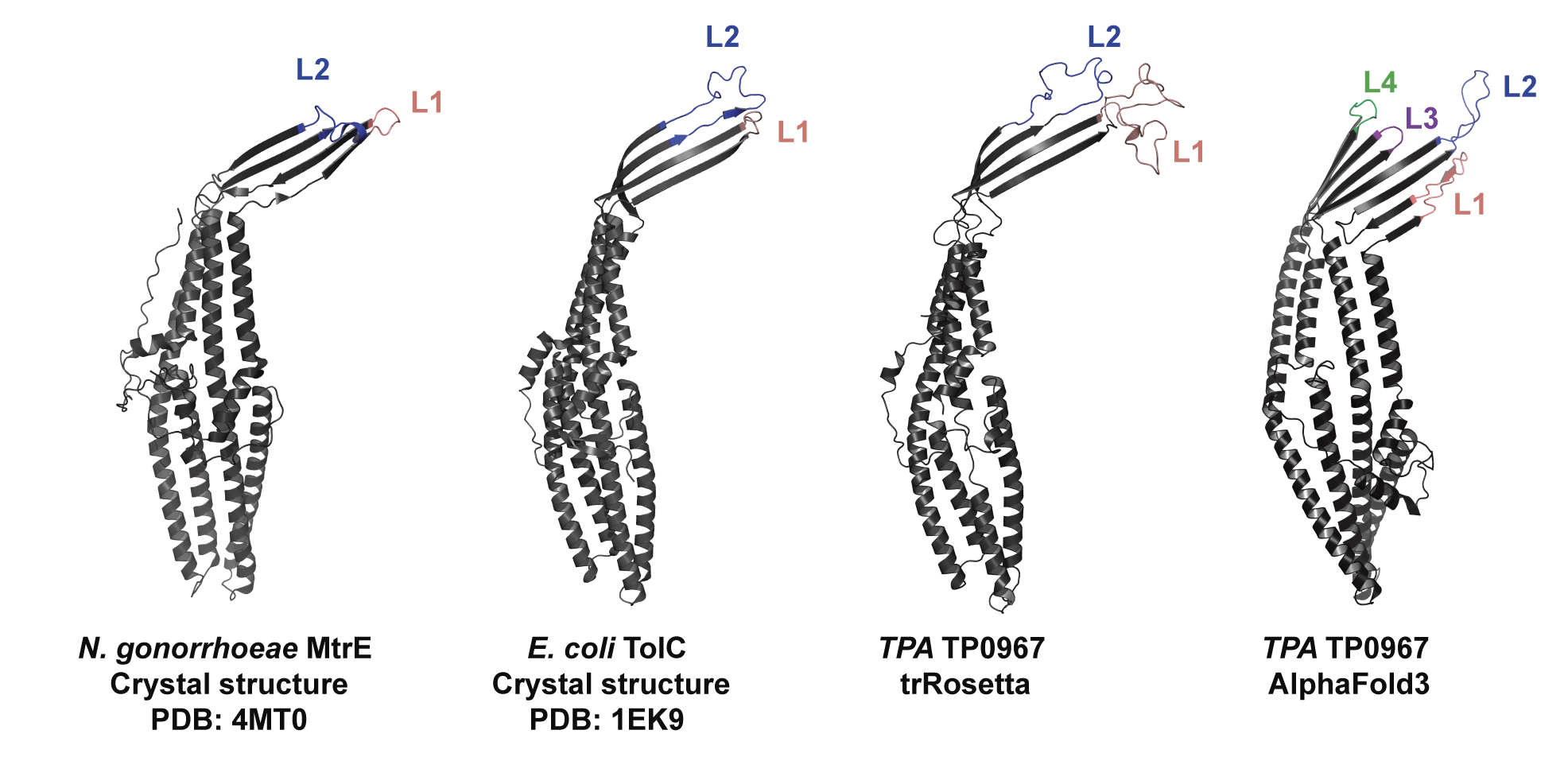

Supplement: S1 Fig — OMF crystal structures of Neisseria gonorrhoeae MtrE and E. coli TolC compared to trRosetta and AlphaFold3 three-dimensional models of OMF TP0967. (PNG) [file ppat.1012443.s001.png]

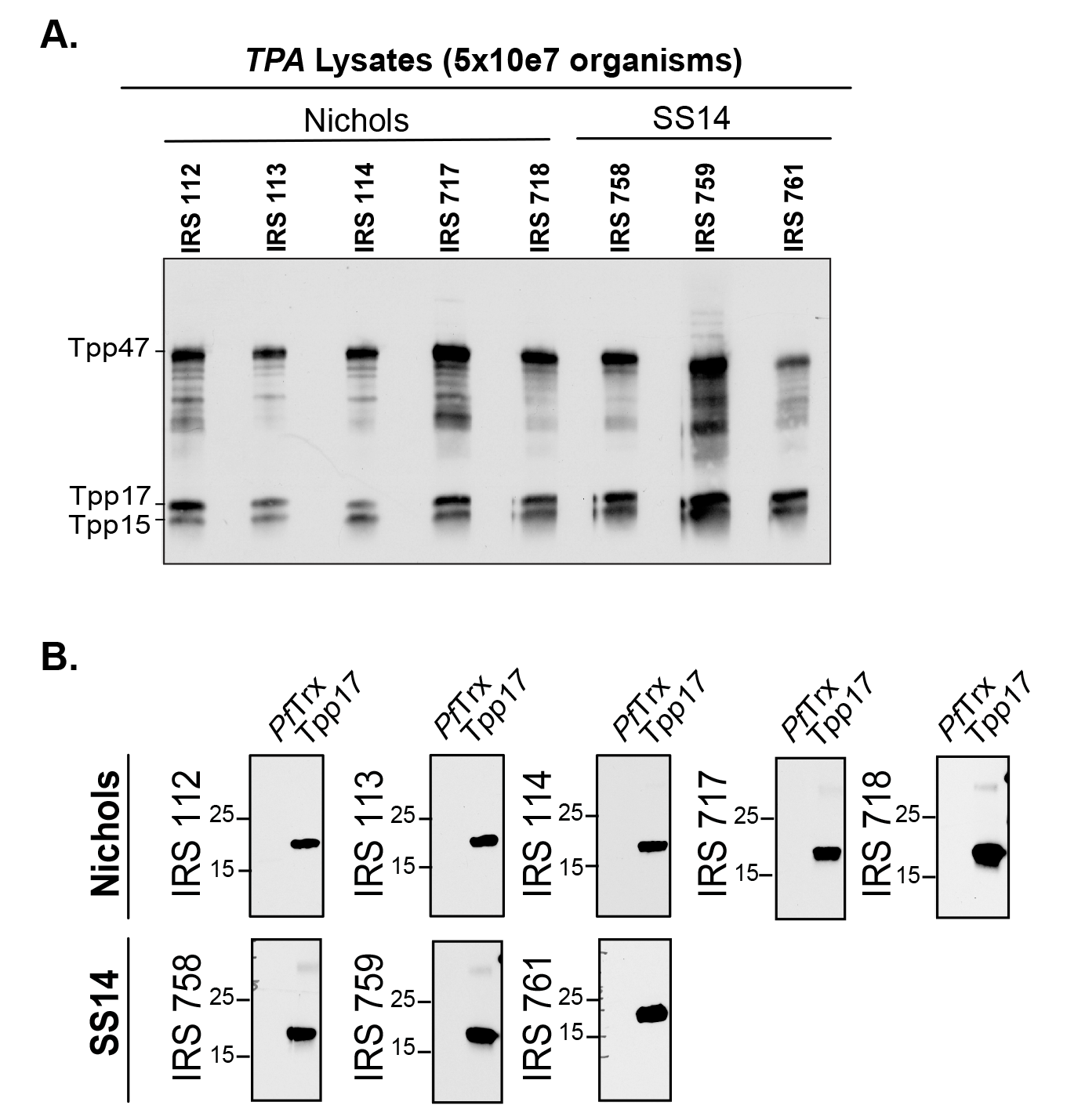

Supplement: S2 Fig — (A) Immunoblot reactivity of Nichols and SS14 IRS with Nichols lysates. (B) Immunoblot reactivity of Nichols and SS14 IRS with PfTrxEmpty and Tpp17 proteins. (PNG) [file ppat.1012443.s002.png]

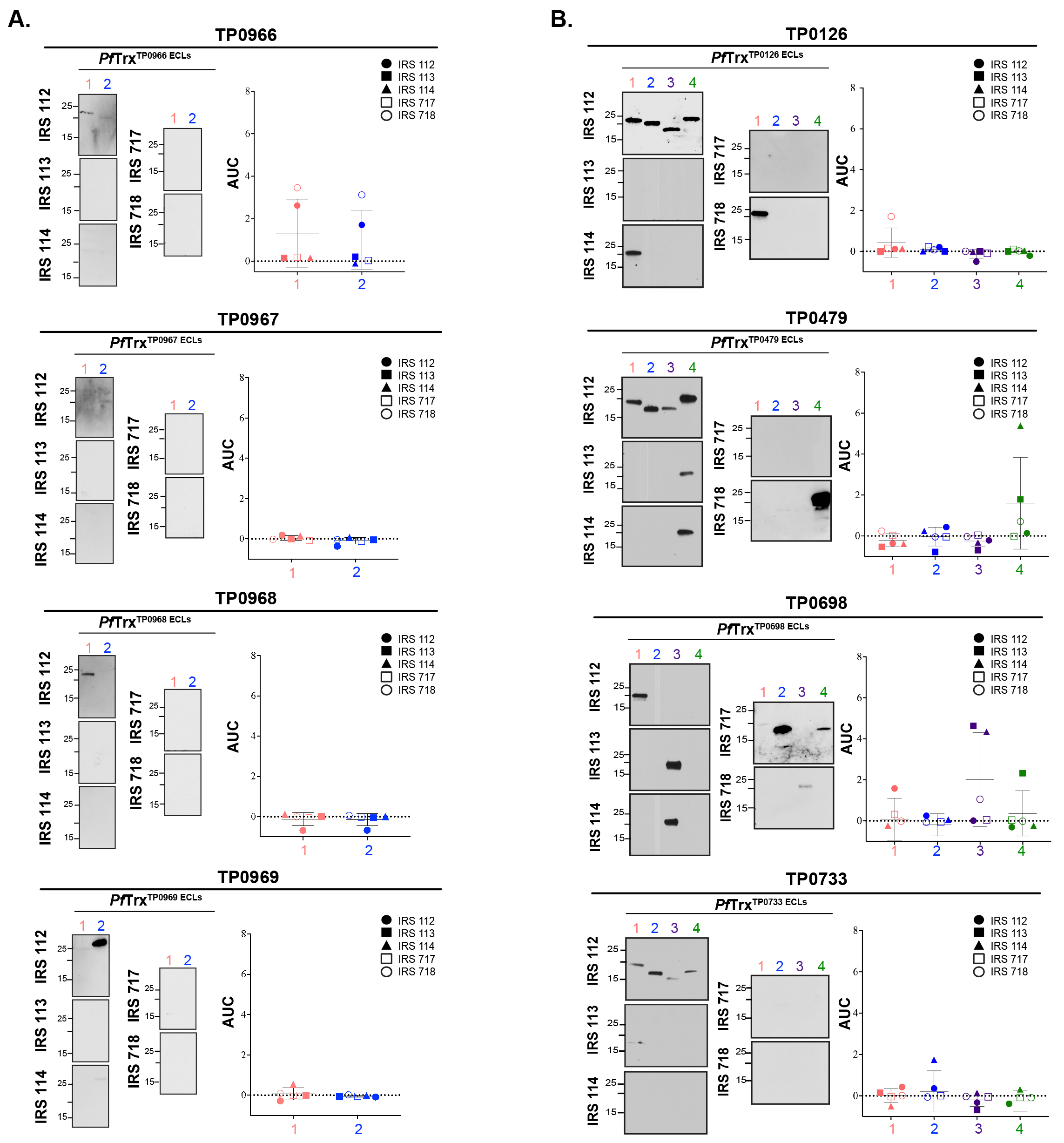

Supplement: S3 Fig — Reactivity by immunoblot (left) and ELISA (right) of scaffolded ECLs of (A) OMFs and (B) 8SβBs against sera from five Nichols immune rabbits. ELISA reactivity was measured as area under the curve (AUC) corrected for PfTrx background (see Methods). n = 3 wells per condition. Data are shown as mean ± SD. Color codes of ECLs are as follows: ECL1-Salmon, ECL2-Blue, ECL3-Purple, ECL4-Green, ECL5-Yellow, ECL6-Cyan, and ECL7-Dark Teal. (PNG) [file ppat.1012443.s003.png]

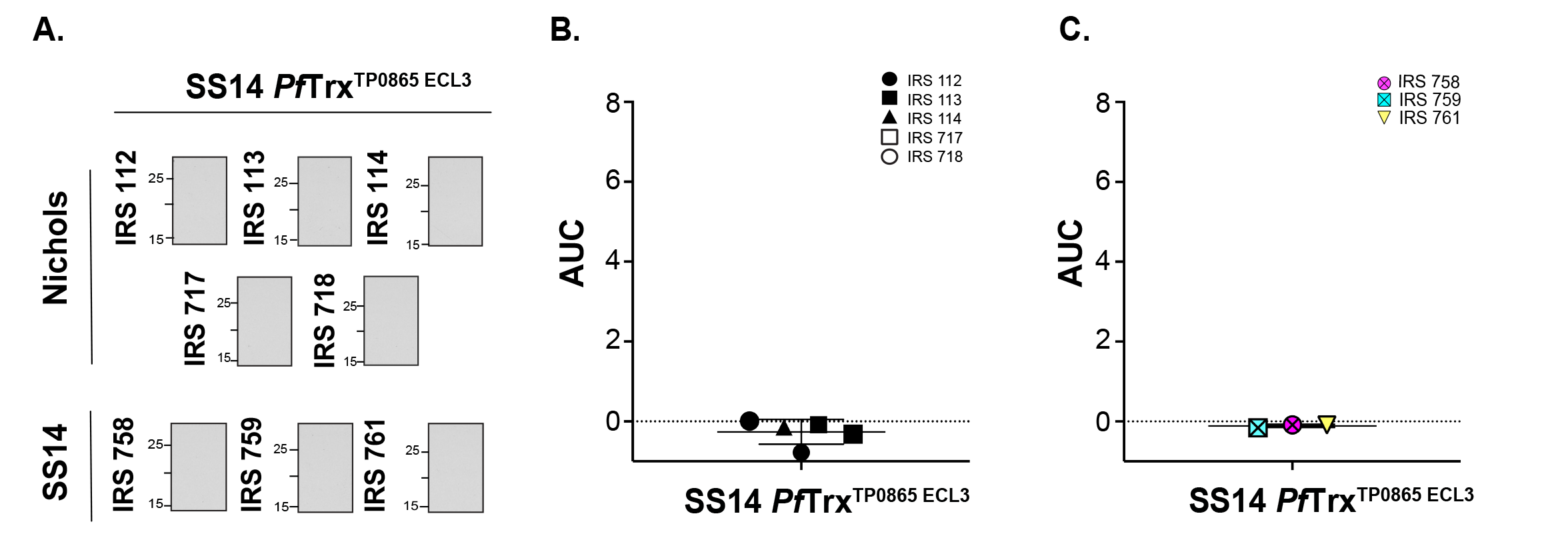

Supplement: S5 Fig — (A) Immunoblot and ELISA (AUC) reactivity of SS14 TP0865 ECL3 with (B) Nichols and (C) SS14 IRS. (PNG) [file ppat.1012443.s005.png]

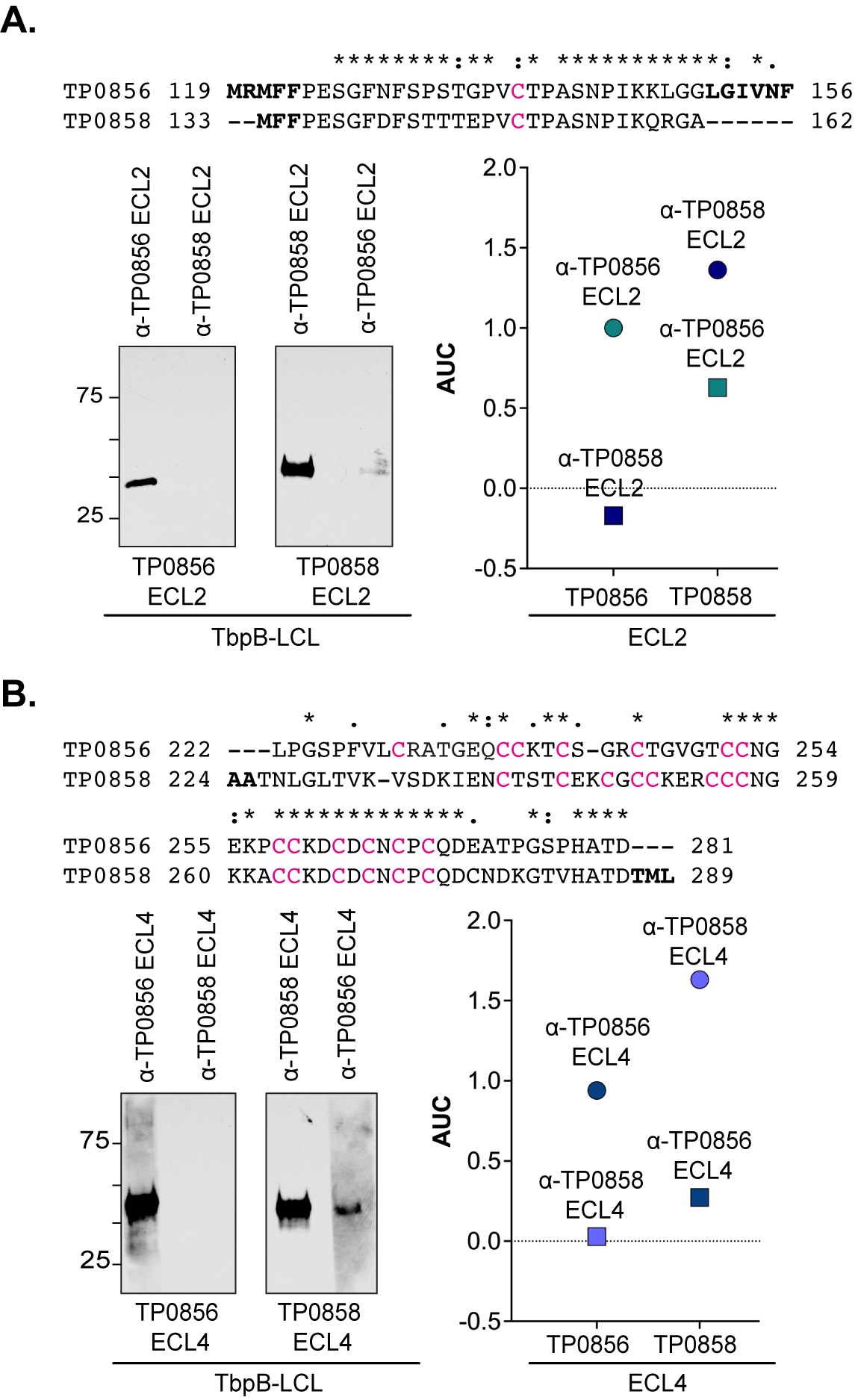

Supplement: S6 Fig — (A) Alignment of TP0856 and TP0858 ECL2 sequences. Immunoblot and ELISA (bottom left and right, respectively) reactivity of rabbit anti-PfTrxTP0856/ECL2 and anti-PfTrxTP0858/ECL2 against TbpB-LCLTP0856/ECL2 and TbpB-LCLTP0858/ECL2. (B) Alignment of TP0856 and TP0858 ECL4 sequences. Immunoblot and ELISA (bottom left and right, respectively) of rabbit anti-PfTrxTP0856/ECL4 and anti-PfTrxTP0858/ECL4 against TbpB-LCLTP0856/ECL4 and TbpB-LCLTP0858/ECL4. (PNG) [file ppat.1012443.s006.png]
