# Supplementary material for: Immunodominant extracellular loops of Treponema pallidum FadL outer membrane proteins elicit antibodies with opsonic and growth-inhibitory activities
Source: PLoS Pathog. 2024 Dec 23;20(12):e1012443. doi: 10.1371/journal.ppat.1012443 (PMC11761103; doi:10.1371/journal.ppat.1012443)
Supplement: S4 Fig — Clustal Omega alignments of the five Nichols FadL orthologs with highlighted variations shown in magenta. Predicted ECLs are indicated using color scheme described in Fig 1. Discontinuous BCE predictions by DiscoTope 2.0 and ElliPro are shown in purple boxes along the sequences. (PDF) [file ppat.1012443.s004.pdf]

TP0865

```
                                                                      ECL1
Nichols  MVRMRRRRACSSGGACGCAAVRGARSFLSVRVLGMRIGMSALCLAPLFARTASLGAWSSQ  60
SS14     MVRMRRRRACSSGGACGCAAVRGARSFLSVRVLGMRIGMSALCLAPLFARTASLGAWSSQ  60
         ************************************************************

Nichols  GGEVLGEVRARVPAHRRVRRAVSGTSVTPVVAMAAKTSEKQKGVGRRALSLRTGGRYEML  120
SS14     GGEVLGEVRARVPAHRRVRRAVSGTSVTPVVAMAAKTSEKQKGVGRRALSLRTGGRYEML  120
         ************************************************************

                                             ECL1
Nichols  GLAFTALADDASFFEANAAGSAAFPYLLVGGFHFARVNQSHTDTIALVHSIGRTGYGFSA  180
SS14     GLAFTALADDASFFEANAAGSAAFPYLLVGGFHFARVNQSHTDTIALVHSIGRTGYGFSA  180
         ************************************************************

              ECL2                                          ECL3
Nichols  SVQYPYLTMEGKAVGGVAIFNVAHRFLSAYRFKGISVGTNVKVGYRDSSAGGERNKK-NQ  239
SS14     SVQYPYLTMEGKTVGGVAIFNVAHRFLSAYRFKGISVGTNVKVGYRDSSAGGERNKKNNQ  240
         ************:******************************************** **

                                                     ECL4
Nichols  GGKKHVVVTADIGLQGTWSVAKNFGSHEPNLWVGGTVKNVGLSVEVDASNSGSSMSGGRT  299
SS14     GGKKHVVVTADIGLQGTWSVAKNFGSHEPNLWVGGTVKNVGLSVEVDASNSGSSMSGGRT  300
         ************************************************************

                                    ECL5
Nichols  VHATNSSFILACAYQPIRWFLFGTGIEWKYNVQEFADNNRFRYGVAFLLLPVQYVAFGSN  359
SS14     VHATNSSFILACAYQPIRWFLFGTGIEWKYNVQEFADNNRFRYGVAFLLLPVQYVAFGSN  360
         ************************************************************

           ECL6                        ECL7
Nichols  VFLTGLASDIRASAGVEFKSTWVRVDLTYTYESDKDEHVISCGIAGFFNRDRRKHLEKEV  419
SS14     VFLTGLASDIRANAGVEFKSTWVRVDLTYTYESDKDEHVISCGIAGFFNRDRRKHLEKEV  420
         *************.**********************************************

Nichols  YTSYLRGLRHYDAQHYEEAIAEWRRTLQRAGSFEPAREGIERATKLLQLNRQVYDFHFLH  479
SS14     YTSYLRGLRHYDAQHYEEAIAEWRRTLQRAGSFEPAREGIERATKLLQLNRQVYDFHFLH  480
         ************************************************************
```

TP0548

```
Nichols  MRQNGAVPMISCSVRRRPRWEPQVGAAFLAFALLPVLASGRGMQAAVATAAG-SSGSGSD  59
SS14     MRQNGAVPMISCSVRRRPRWEPQVGAAFLAFALLPVLASGRGMQAAVATAAGGSSGCSDN  60
         **************************************************** ***...:

                                                                ECL1
Nichols  GKHPGKEQFLQFLIPSGGRYEYLGVSFTALADDASFFEANPAGSAGLSRGEVALFHHSQI  119
SS14     GNHPGKEQFLQFLIPSGGRYEYLGVSFTALADDASFFEANPAGSAGLSRGEVALFHHSQI  120
         *:**********************************************************

         ECL1                                   ECL2
Nichols  HDSHTETVSFARRTQNTGYGASVRAFSSESDLKSFFGG---NSGGNKNGGHQGKQGKGFV  176
SS14     HDSHTETVSFARRTQNTGYGASVRAFSSESDLKSFYGMIGSSSSGKNGGHQGKQGKGFV  180
         ***********************************:*    .*...**************

                                      ECL3
Nichols  AIANASHTFCGQYRFKGVSFGCNFKMGFRKGKTDSHVTVAGDLGLRAAFSVAKNFGSNEP  236
SS14     AIANASHTFCGQYRFKGVSFGCNFKMGFRKGKTDSHVTVAGDLGLRAAFSVAKNFGSNEP  240
         ************************************************************

                  ECL4                                      ECL5
Nichols  NMHVGLVLKNAGISVKTNSCQVEHLNPAIAVGFAYRPVYAFLFSLGLQQTLTKRESPVCS  296
SS14     NMHVGLVLKNAGISVKTKDSDVKHLNPAIAVGFAYRPVYAFLFSLGLQQTLTKKELPVCS  300
         *****************:..:*:******************************:* ****

                             ECL6                           ECL7
Nichols  VGFMFFCTQHVTLLASAACEGGAYALSGGAEIRIGSFHLDMGYRYDQIFQAAHPHHVSVG  356
SS14     VGFMFFCTHQVTLLASAACKGKAYALSGGAEIRIGSFHLDMGYRYDQIFQAAHPHHVSVG  360
         ********::*********:* **************************************

Nichols  LKWLIPNGGTQADQALLVKESYLVGLRFYDQRRYQEAITAWQLTLRQDPGFEPAAEGIER  416
SS14     LKWLIPNGGTQADQALLVKESYLVGLRFYDQRRYQEAITAWQLTLRQDPGFEPAAEGIER  420
         ************************************************************

Nichols  ARRFLKLHEKLSLFDILN  424
SS14     ARRFLKLHEKLSLFDILN  438
         ******************
```

TP0856

```
Nichols MVHYKSVFYKSAALVCGFVLAGASVAIASSEAAAKTRSKMSEFKRRAVSSPSGGRLSVLD 60
SS14    MVHYKSVFYKSAALVCGFVLAGASVAIASSEAAAKTRSKMSEFKRRAVSSPSGGRLSVLD 60
        ************************************************************

        ECL1
Nichols GSFTALANDASFFEANPAGSANMTHSELTFAHTVGFNNSHAETLSYVGQSGNWGYGASMR 120
SS14    GSFTALANDASFFEANPAGSANMTHSELTFAHTVGFNNSHAETLSYVGQSGNWGYGASMR 120
        ************************************************************

        ECL2 ECL3
Nichols MFFPESGFNFSPSTGPVCTPASNPIKKLGGLGIVNFSRRFGGLSIGANLKAGFRDAQGLT 180
SS14    MFFPESGFNFSPSTGPVCTPASNPIKKLGGLGIVNFSRRFGGLSIGANLKAGFRDAQGLT 180
        ************************************************************

        ECL4
Nichols HLSLGTDVGLQWVGNVAKFFSSAEPNMYVGLSATNLGFTVKLPGSPFVLCRATGEQCCKT 240
SS14    HLSLGTDVGLQWVGNVAKFFSSAEPNMYVGLSATNLGFTVKLPGSPFVLCRATGEQCCKT 240
        ************************************************************

        ECL4
Nichols CSGRCTGVGTCCNGEKPCCKDCDCNCPCQDEATPGSPHATDTMLRAGFAYRPLSWFLFSV 300
SS14    CSGRCTGVGTCCNGEKPCCKDCDCNCPCQDEATPGSPHATDTMLRAGFAYRPLSWFLFSV 300
        ************************************************************

        ECL5 ECL6
Nichols GVATRVNVSNLQVDHLWKRSSYALGMILDPVRFLTLLSGVAVNANGKVRAGVGAEIRVAC 360
SS14    GVATRVNVSNLQVDHLWKRSSYALGMILDPVRFLTLLSGVAVNANGKVRAGVGAEIRVAC 360
        ************************************************************

        ECL7
Nichols FQVSASYRYDSTGDEQQGTPHNMSLGASILLGRK 394
SS14    FQVSASYRYDSTGDEQQGTPHNMSLGASILLGRK 394
        **********************************
```

TP0858

```
Nichols MLRLPTARACITMGTMIRHTFTHRCGALLCALALGSSTMAATAAAKPKKGQMQKLRQRPV 60
SS14    MLRLPTARACITMGTMIRHTFTHRCGALLCALALGSSTMAATAAAKPKKGQMQKLRQRPV 60
        ************************************************************

        ECL1
Nichols WAPTGGRYASLDGAFTALANDASFFEANPAGSANMTHGELAFFHTTGFGSFHAETLSYVG 120
SS14    WAPTGGRYASLDGAFTALANDASFFEANPAGSANMTHGELAFFHTTGFGSFHAETLSYVG 120
        ************************************************************

        ECL2
Nichols QSGNWGYGASMRMFFPESGFDFSTTTEPVCTPASNPIKQRGAIGIINFARRIGGLSLGAN 180
SS14    QSGNWGYGASMRMFFPESGFDFSTTTEPVCTPASNPIKQRGAIGIINFARRIGGLSLGAN 180
        ************************************************************

        ECL3 ECL4
Nichols LKAGFRDAQGLQHTSVSSDIGLQWVGNVAKSFTSEEPNLYIGLAATNLGLTVKVSDKIEN 240
SS14    LKAGFRDAQGLQHTSVSSDIGLQWVGNVAKSFTSEEPNLYIGLAATNLGLTVKVSDKIEN 240
        ************************************************************

        ECL4
Nichols CTSTCEKCGCCKERCCCNGKKACCKDCDCNCPCQDCNDKGTVHATDTMLRAGFAYRPFSW 300
SS14    CTSTCEKCGCCKERCCCNGKKACCKDCDCNCPCQDCNDKGTVHATDTMLRAGFAYRPFSW 300
        ************************************************************

        ECL5 ECL6
Nichols FLFSLGATTSMNVQTLASSDAKSLYQNLAYSIGAMFDPFSFLSLSSSFRINHKANMRVGV 360
SS14    FLFSLGATTSMNVQTLASSDAKSLYQNLAYSIGAMFDPFSFLSLSSSFRINHKANMRVGV 360
        ************************************************************

        ECL7
Nichols GAEARIARIKLNAGYRCDVSDISSGSGCTGAKASHYLSLGGAILLGRN 408
SS14    GAEARIARIKLNAGYRCDVNDISSGSGCTGAKASHYLSLGGAILLGRN 408
        *******************.****************************
```

TP0859

```
Nichols LVRRPCVSAAPVRVGGRLVFGFARVGSRGLCLGALLLSPRIVLAQHVADAPLGARGVVPR 60
SS14    MVRRPCVSAAPVRVGGRLVFGFARVGSRGLCLGALLLSPRIVLAQHVADAPLGARGVVPR 60
        :***********************************************************

Nichols SSLPRRTRAARATTLRSRGGVVSSRASGGTLVVTAQKPKVMARNDVDYRPLSLQAGGRQG 120
SS14    SSLPRRTRAARATTLRSRGGVVSSRASGGTLVVTAQKPKVMARNDVDYRPLSLQAGGRQG 120
        ************************************************************

        ECL1
Nichols SLDLVATATADDASFFEANAAGSATIPRMTLAFFHTMRISDSHIDVLSFVGRAGRTGYGV 180
SS14    SLDLVATATADDASFFEANAAGSATIPRMTLAFFHTMRISDSHIDVLSFVGRAGRTGYGV 180
        ************************************************************

        ECL2 ECL3
Nichols SARAFYPDMSSKTTGFVGIFNVSHAFSSAYRFKGVSVGANLKVGYRHTRGGGSSQSKSSN 240
SS14    SARAFYPDMSSKTTGFVGIFNVSHAFSSAYRFKGVSVGANLKVGYRHTRGGGSSQSKSSN 240
        ************************************************************

        ECL4
Nichols GKENHHIVLTADVGVRGAWTVSKNFGAHEPNLWAGVAFRNIGASINATNLHGNNGAGGSG 300
SS14    GKENHHIVLTADVGVRGAWTVSKNFGAHEPNLWAGVAFRNIGASINATNLHGNNGAGGSG 300
        ************************************************************

        ECL4 ECL5
Nichols GGGGGNGDGKPAHVTDSRVILALAYQPVRYFLFGAGLEWLYNVGSIKAVNSLRYGAAFML 360
SS14    GGGGGNGDGKPAHVTDSRVILALAYQPVRYFLFGAGLEWLYNVGSIKAVNSLRYGAAFML 360
        ************************************************************

        ECL6 ECL7
Nichols FPLRQLAFSSSVVMKGMGPQQVRASAGAEVQFSHVRCTASYSYLWSATPTRPHYVSIGVA 420
SS14    FPLRQLAFSSSVVMKGMGPQQVRASAGAEVQFSHVRCTASYSYLWSATPTRPHYVSIGVA 420
        ************************************************************

Nichols GFLKPVPEQPLWQEVYRSYLRGLRHYHAQRYAEAIAEWKRTLQQGVSFEPAREGIERATK 480
SS14    GFLKPVPEQPLWQEVYRSYLRGLRHYHAQRYAEAIAEWKRTLQQGVSFEPAREGIERATK 480
        ************************************************************

Nichols LLQLNQKVHDFNIFT 495
SS14    LLQLNQKVHDFNIF- 494
        **************
```
