# Supplementary material for: Immunodominant extracellular loops of Treponema pallidum FadL outer membrane proteins elicit antibodies with opsonic and growth-inhibitory activities
Source: PLoS Pathog. 2024 Dec 23;20(12):e1012443. doi: 10.1371/journal.ppat.1012443 (PMC11761103; doi:10.1371/journal.ppat.1012443)
Supplement: S1 Table — (PDF) [file ppat.1012443.s007.pdf]

Family	Protein	ECL	Name	Amino Acid Sequence	Primers
	TP0966	ECL1	*Pf*Trx$^{TP0966/ECL1}$	NLNGNGAGGMANGTPTLSPYVHLFFPTYQNLSLKADIAIKT	FW - GGGATGCGGTGGATCCCTGAATGGGAACGGGGCC<br>RV - GACGACACGGGGATCCGGTCTTGATCGCAATATCCGC
	TP0966	ECL2	*Pf*Trx$^{TP0966/ECL2}$	EGKSLKRILANVRMDFPGGGFWLGLNLPYPQWSRVE	FW - GGGATGCGGTGGATCCCTGAATGGGAACGGGGCC<br>RV - GACGACACGGGGATCCGGTCTTGATCGCAATATCCGC
	TP0967	ECL1	*Pf*Trx$^{TP0967/ECL1}$	AWNADGVKFRITPKASVAFPSFYNLTTHFGMTVTQPNGAAGGGGGGGGGDWQKT	FW - GGGATGCGGTGGATCCCTGAATGGGAACGGGGCC<br>RV - GACGACACGGGGATCCGGTCTTGATCGCAATATCCGC
OMF	TP0967	ECL2	*Pf*Trx$^{TP0967/ECL2}$	TLDEGTGELSVAFPSVKITSALAIGYTGTLSI	FW - GGGATGCGGTGGATCCCTGAATGGGAACGGGGCC<br>RV - GACGACACGGGGATCCGGTCTTGATCGCAATATCCGC
	TP0968	ECL1	*Pf*Trx$^{TP0968/ECL1}$	IRGHSAGDFGLPRFGIKPIIGVRSPRYNNLVVSIDTARVTSIGNIS	FW - GGGATGCGGTGGATCCCTGAATGGGAACGGGGCC<br>RV - GACGACACGGGGATCCGGTCTTGATCGCAATATCCGC
	TP0968	ECL2	*Pf*Trx$^{TP0968/ECL2}$	MGTRKTFILFKGDGTESLEGSGTVALHMPSVNAQVEVKVPYAERGKHSRDKVG	FW - GGGATGCGGTGGATCCCTGAATGGGAACGGGGCC<br>RV - GACGACACGGGGATCCGGTCTTGATCGCAATATCCGC
	TP0969	ECL1	*Pf*Trx$^{TP0969/ECL1}$	LFNKKNGNGANGYKVEMAPHLSIASPFIGNSRLNLVAPRKLDGVTSTSTV	FW - GGGATGCGGTGGATCCCTGAATGGGAACGGGGCC<br>RV - GACGACACGGGGATCCGGTCTTGATCGCAATATCCGC
	TP0969	ECL2	*Pf*Trx$^{TP0969/ECL2}$	TDGEENKQLTNGMAPAAPSTTSTYGGTFNMAFPGGDSSFTVQNSKGLAGI	FW - GGGATGCGGTGGATCCCTGAATGGGAACGGGGCC<br>RV - GACGACACGGGGATCCGGTCTTGATCGCAATATCCGC
	TP0126	ECL1	*Pf*Trx$^{TP0126/ECL1}$	PLFQVDWCNSGRGDDRNANAQTNGHKYIYPAFS	FW - GGGATGCGGTGGATCCCCGCTGTTCCAGGTTGATTGG<br>RV - GACGACACGGGGATCCGCTAAACGCCGGATAGATATACTTG
	TP0126	ECL2	*Pf*Trx$^{TP0126/ECL2}$	SVQYHCSYPNNTYSPTTPYYYLAIPV	FW - GGGATGCGGTGGATCCAGCGTTCAGTATCACTGCAGC<br>RV - GACGACACGGGGATCCCACCGGAATCGCCAGATAATAG
	TP0126	ECL3	*Pf*Trx$^{TP0126/ECL3}$	QHYYTSTYYGL	FW - CACCTATTATGGTCTGGGATCCCCGTGTCGTCTG<br>RV - CTGGTGTAATAGTGCTGACCGCATCCCGGAATGCT
	TP0126	ECL4	*Pf*Trx$^{TP0126/ECL4}$	ATYSGVPRSCEKIIEEDRQQTNTRTAQF	FW - GAAGATCGTCAGCAAACCAATACCCGTACCGCGCAGTTTGGATCCCCGTGTCGTCTG<br>RV - CTCAATGATTTTTTCGCAGCTACGCGGAACGCCGCTGTAACCGCATCCCGGAATGCT
	TP0479	ECL1	*Pf*Trx$^{TP0479/ECL1}$	VGAHPWGKEPAPRTDVLLYTP	FW - AGAACCGGCGCCGCGTACCGACGTTCTGCTGTATACCCCGTCCCCGTGTCGTCTGGTTG<br>RV - CGCGGCGCCGGTTCTTTACCCCACGGATGCGCACCAACTCCACCGCATCCCGGAATG
	TP0479	ECL2	*Pf*Trx$^{TP0479/ECL2}$	AGLAFLMFRA	FW - GTTCCTGATGTTTCGTGGATCCCCGTGTCGTCTG<br>RV - GCCAGACCCGCATCCAGACCGCATCCCGGAATGCT
	TP0479	ECL3	*Pf*Trx$^{TP0479/ECL3}$	LSTQDDKLVGIP	FW - CGATAAGCTGGTTGGTGGATCCCCGTGTCGTCTG<br>RV - TCTTGGGTGCTCAGAATACCGCATCCCGGAATGCT
8SβB	TP0479	ECL4	*Pf*Trx$^{TP0479/ECL4}$	GSGVNVPLTKNLKKGANGNGQDLTWSNIWAKYCCTSLVM	FW - GGGATGCGGTGGATCCGGCAGCGGTGTGAACGTTCC<br>RV - GACGACACGGGGATCCCATAACCAGGCTGGTGCAGC
	TP0698	ECL1	*Pf*Trx$^{TP0698/ECL1}$	GITSVYQFGSNGGSDGTSSGKGVSFDRLIGRVD	FW - GGGATGCGGTGGATCCGGTATTACCAGCGTGTACCAATTCG<br>RV - GACGACACGGGGATCCATCCACACGACCGATCAGACG
	TP0698	ECL2	*Pf*Trx$^{TP0698/ECL2}$	SSLTNVFVRA	FW - TGACCAATGTCTTTGTGCGTGCATCCCCGTGTCGTCTGGTTG<br>RV - CAAAGACATTGGTCAACGAACTTCCACCGCATCCCGGAATG
	TP0698	ECL3	*Pf*Trx$^{TP0698/ECL3}$	GVNICGDSCATSEGKSSAWYSKLLYSVPLN	FW - GGGATGCGGTGGATCCGGTGTTAACATTTGCGGTGACAGC<br>RV - GACGACACGGGGATCCGTTCAGCGGCACGCTATACAGC
	TP0698	ECL4	*Pf*Trx$^{TP0698/ECL4}$	STAVGVRDFNFKEFTLPLSL	FW - ATTTCAACTTTAAGGAGTTTACCCTGCCGCTGAGCCTGTCCCCGTGTCGTCTGGTTG<br>RV - CCTTAAAGTTGAAATCACGAACGCCCACCGCGGTGCTTCCACCGCATCCCGGAATG
	TP0733	ECL1	*Pf*Trx$^{TP0733/ECL1}$	GCQLYIAGGNGTNGSSSSGTNGNGNGKLLGGG	FW - GGGATGCGGTGGATCCTACATAGCAGGGGGAAATGGAACC<br>RV - GACGACACGGGGATCCCCCGCCCCCGAGCAGTTT
	TP0733	ECL2	*Pf*Trx$^{TP0733/ECL2}$	FECYRTTGSNYYFSVPI	FW - GACCGGGTCAAACTATTACTTTTCTGTTCCCATCTCCCCGTGTCGTCTGGTTG<br>RV - TAGTTTGACCCGGTCGTGCGGTAACACTCAAATCCACCGCATCCCGGAATG
	TP0733	ECL3	*Pf*Trx$^{TP0733/ECL3}$	LNIQSYLSKKAPGLI	FW - GAAGGCGCCGGGGCTTGGATCCCCGTGTCGTCTG<br>RV - TTGCTGAGATAGGACTGACCGCATCCCGGAATGCT
	TP0733	ECL4	*Pf*Trx$^{TP0733/ECL4}$	YTQLGDIASSPDKCRAVGLA	FW - TGCAAGCTCCCCCGACAAGTGCAGAGCCGTGGGCCTTTCCCCGTGTCGTCTGGTTG<br>RV - TCGGGGGAGCTTGCAATGTCCCCAAGCTGCGTGTATCCACCGCATCCCGGAATG
	TP0548	ECL1	*Pf*Trx$^{TP0548/ECL1}$	QIHDSH	FW - AGATTCACGACAGCCACTCCCCGTGTCGTCTGGTTG<br>RV - GGCTGTCGTGAATCTGTCCACCGCATCCCGGAATG
	TP0548	ECL2	*Pf*Trx$^{TP0548/ECL2}$	FSSESDLKSFFGGNSGGNKNGGHQGKQGKGFVAIA	FW - GGGATGCGGTGGATCCAGCGAGAGCGATCTGAAGAGC<br>RV - GACGACACGGGGATCCCGCAATCGCCACGAAGCC
	TP0548	ECL3	*Pf*Trx$^{TP0548/ECL3}$	MGFRKGKTDSHVTV	FW - TAAGGGCAAAACCGACAGCCACGTGACCTCCCCGTGTCGTCTGGTTG<br>RV - TCGGTTTTGCCCTTACGGAAACCCATTCCACCGCATCCCGGAATG
	TP0548	ECL4	*Pf*Trx$^{TP0548/ECL4}$	KNAGISVKTNSCQVEHLNPA	FW - AAACCAACAGCTGCCAGGTTGAGCACCTGAACCCGTCCCCGTGTCGTCTGGTTG<br>RV - GGCAGCTGTTGGTTTTCACGCTGATGCCCGCGTTTCCACCGCATCCCGGAATG
	TP0548	ECL5	*Pf*Trx$^{TP0548/ECL5}$	QTLTKRESPVC	FW - GACCAAACGTGAAAGCCCGGTGTGCTCCCCGTGTCGTCTGGTTG<br>RV - CTTTCACGTTTGGTCAGGGTTTGTCCACCGCATCCCGGAATG
	TP0548	ECL6	*Pf*Trx$^{TP0548/ECL6}$	ACEGGAYA	FW - GCGAGGGTGGCGCGTACGCGTCCCCGTGTCGTCTGGTTG<br>RV - ACGCGCCACCCTCGCACGCTCCACCGCATCCCGGAATG
	TP0548	ECL7	*Pf*Trx$^{TP0548/ECL7}$	YDQIFQAAHPH	FW - GATCTTTCAGGCGGCGCACCCGCACTCCCCGTGTCGTCTGGTTG<br>RV - GCCGCCTGAAAGATCTGATCATATCCACCGCATCCCGGAATG
	TP0548	Hatch	*Pf*Trx$^{TP0548/Hatch}$	SGRGMQAAVATAAGSSGSGSDGKHPGKEQFLQFLIPSGGRYEYLGVSFTALADDASFFEANPAGSAGLSRG	FW - GGGATGCGGTGGATCCAGCGGCCGTGGTATGCAGG<br>RV - GACGACACGGGGATCCGCTCGGGATCAGAAATTGCAGG
	TP0856	ECL1	*Pf*Trx$^{TP0856/ECL1}$	AHTVGFNNSHAETLSYV	FW - TAACAACAGCCACGCGGAGACCCTGAGCTACGTTGGATCCCCGTGTCGTCTGGT<br>RV - GCGTGGCTGTTGTTAAAGCCCACGGTGTGCGCGGATCCACCGCATCCCGG
	TP0856	ECL2	*Pf*Trx$^{TP0856/ECL2}$	MRMFFPESGFNFSPSTGPVCTPASNPIKKLGGLGIVNF	FW - GGGATGCGGTGGATCCATGCGTATGTTCTTTCCGGAAAGC<br>RV - GACGACACGGGGATCCGAAGTTAACAATACCCAGGCCACCC
	TP0856	ECL3	*Pf*Trx$^{TP0856/ECL3}$	GFRDAQGLTHLSLG	FW - CAGACCCTGCGCATCACGGAAACCGGATCCACCGCATCCCGG<br>RV - GATGCGCAGGGTCTGACCCACCTGAGCGGATCCCCGTGTCGTCTGGT
	TP0856	ECL4	*Pf*Trx$^{TP0856/ECL4}$	LGFTVKLPGSPFVLCRATGEQCCKTCSGRCTGVGTCCNGEKPCCKDCDCNCPCQDEATPGSPHATDTML	FW - GGGATGCGGTGGATCCCTGCCGGGCAGCCCG<br>RV - GACGACACGGGGATCCCAGCATGGTATCGGTCGCGT
	TP0856	ECL5	*Pf*Trx$^{TP0856/ECL5}$	TRVNVSNLQVDHLWKRS	FW - AGCGACGCGAAGAGCCTGTACCAAAACCTGGCGTATAGCGGATCCCCGTGTCGTCTGGTTGAACG<br>RV - GCTCTTCGCGTCGCTGCTCGCCAGGGTCTGAACGTTCATGCTGGTGGATCCACCGCATCCCGGAATGCTAAA
	TP0856	ECL6	*Pf*Trx$^{TP0856/ECL6}$	AVNANGKVRAGV	FW - AGTGCGTGCGGGTGTTGGATCCCCGTGTCGTCTGGT<br>RV - ACACCCGCACGCACTTTGCCGTTCGCGTTAACGGATCCACCGCATCCCGG
	TP0856	ECL7	*Pf*Trx$^{TP0856/ECL7}$	DSTGDEQQGTPHN	FW - ACAGCAAGGTACCCCGCACAACGGATCCCCGTGTCGTCTGGT<br>RV - GGGGTACCTTGCTGTTCATCACCGGTGCTGTCGGATCCACCGCATCCCGG
	TP0856	Hatch	*Pf*Trx$^{TP0856/Hatch}$	SSEAAAKTRSKMSEFKRRAVSSPSGGRLSVLDGSFTALANDASFFEANPAGSANMTH	FW - GCGGCGGCGAAGACCCGTAGCAAAATGGGATCCCCGTGTCGTCTGGT<br>RV - GGTCTTCGCCGCCGCCTCGCTGCTGGATCCACCGCATCCCGG
	TP0858	ECL1	*Pf*Trx$^{TP0858/ECL1}$	FHTTGFGSFHAE	FW - GCTGCCGAAACCGGTGGTGTGAAAGGATCCACCGCATCCCGG<br>ACCGGTTTCGGCAGCTTTCACGCGGAAGGATCCCCGTGTCGTCTGGT
	TP0858	ECL2	*Pf*Trx$^{TP0858/ECL2}$	MFFPESGFDFSTTTEPVCTPASNPIKQRGA	FW - GGGATGCGGTGGATCCAACTGGGGTTATGGCGCGAG<br>RV - GACGACACGGGGATCCCGCACCACGTTGCTTGATCG
	TP0858	ECL3	*Pf*Trx$^{TP0858/ECL3}$	GFRDAQGLQHTSV	FW - CAGACCCTGCGCGTCACGAAAGCCGGATCCACCGCATCCCGG<br>RV - GACGCGCAGGGTCTGCAACACACCAGCGGATCCCCGTGTCGTCTGGT
	TP0858	ECL4	*Pf*Trx$^{TP0858/ECL4}$	AATNLGLTVKVSDKIENCTSTCEKCGCCKERCCCNGKKACCKDCDCNCPCQDCNDKGTVHATDTML	FW - GGGATGCGGTGGATCCGCGGCGACCAACCTGGG<br>RV - GACGACACGGGGATCCCAGCATGGTATCGGTCGCGT
FadL	TP0858	ECL5	*Pf*Trx$^{TP0858/ECL5}$	SMNVQTLASSDAKSLYQNLA	FW - CGAGCAGCGACGCGAAGAGCCTGTACCAAAACTCCCCGTGTCGTCTGGTTG<br>RV - TCGCGTCGCTGCTCGCCAGGGTCTGAACGTTTCCACCGCATCCCGGAATG

TP0858	ECL6	*Pf*Trx$^{\text{TP0858/ECL6}}$	SFRINHKANMRV	FW - CGCCTTGTGGTTAATACGAAAGCTGGATCCACCGCATCCCGG<br>RV - ATTAACCACAAGGCGAACATGCGTGTGGGATCCCCGTGTCGTCTGGT
TP0858	ECL7	*Pf*Trx$^{\text{TP0858/ECL7}}$	RCDVSDISSGSGCTGAKASHY	FW - GCAGCCGCTACCGCTGCTGATATCGCTCACGTCGCAACGGGATCCACCGCATCCCGG<br>RV - AGCGGTAGCGGCTGCACCGGTGCGAAAGCGAGCCACTATGGATCCCCGTGTCGTCTGGT
TP0858	Hatch	*Pf*Trx$^{\text{TP0858/Hatch}}$	AAAKPKKGQMQKLRQRPVWAPTGGRYASLDGAFTALANDASFFEANPAGSANMTH	FW - GGTCAAATGCAAAAACTGCGTCAACGTCCGGTGGGATCCCCGTGTCGTCTGGTTGAACG<br>RV - GTTTTTGCATTTGACCTTTCTTCGGCTTCGCCGCCGCGGATCCACCGCATCCCGGAATGCTAAA
TP0859	ECL1	*Pf*Trx$^{\text{TP0859/ECL1}}$	MRISDSH	FW - TGCGTATCAGCGACAGCTCCCCGTGTCGTCTGGTTG<br>RV - TGTCGCTGATACGCATTCCACCGCATCCCGGAATG
TP0859	ECL2	*Pf*Trx$^{\text{TP0859/ECL2}}$	FYPDMSSKTTGFV	FW - TGAGCAGCAAGACCACCGGCTTCGTGTCCCCGTGTCGTCTGGTTG<br>RV - TGGTCTTGCTGCTCATGTCCGGATATCCACCGCATCCCGGAATG
TP0859	ECL3	*Pf*Trx$^{\text{TP0859/ECL3}}$	GYRHTRGGGSSQSKSSNGKENHHIVLT	FW - GGGATGCGGTGGATCCTATCGTCACACCCGTGGT<br>RV - GACGACACGGGGATCCCACAATGTGGTGGTTCTCTTTACCG
TP0859	ECL4	*Pf*Trx$^{\text{TP0859/ECL4}}$	FRNIGASINATNLHGNNGAGGSGGGGGGNGDGKPAHVTDS	FW - GGGATGCGGTGGATCCTTTCGTAACATCGGTGCGAGC<br>RV - GACGACACGGGGATCCGCTATCGGTCACGTGCGC
TP0859	ECL5	*Pf*Trx$^{\text{TP0859/ECL5}}$	LYNVGSIKAVNSL	FW - GGGCAGCATTAAGGCGGTTAACAGCCTGTCCCCGTGTCGTCTGGTTG<br>RV - GCCTTAATGCTGCCCACGTTGTACAGTCCACCGCATCCCGGAATG
TP0859	ECL6	*Pf*Trx$^{\text{TP0859/ECL6}}$	VMKGMGPQQVR	FW - AGGCATGGGTCCGCAACAAGTTCGTTCCCCGTGTCGTCTGGTTG<br>RV - TGCGGACCCATGCCTTTCATAACTCCACCGCATCCCGGAATG
TP0859	ECL7	*Pf*Trx$^{\text{TP0859/ECL7}}$	YLWSATPTRPHY	FW - GCGCGACCCCGACCCGTCCGCACTACTCCCCGTGTCGTCTGGTTG<br>RV - GGGTCGGGGTCGCGCTCCACAGATATCCACCGCATCCCGGAATG
TP0859	Hatch	*Pf*Trx$^{\text{TP0859/Hatch}}$	QHVADAPLGARGVVPRSSLPRRTRAARATTLRSRGGVVSSRASGGTLVVTAQKPKVMARNDVDYRPLSLQAGGRQGSLDLVATATADDASFFEANAAGSATIPR	FW - GGGATGCGGTGGATCCCAACATGTTGCGGATGCGC<br>RV - GACGACACGGGGATCCCAGGCTCAGCGGACGATAATC
TP0865	ECL1	*Pf*Trx$^{\text{TP0865/ECL1}}$	ARVNQSH	FW - CCCGGGTTAATCAATCGTCCCCGTGTCGTCTGGTTG<br>RV - ATTGATTAACCCGGGCTCCACCGCATCCCGGAATG
TP0865	ECL2	*Pf*Trx$^{\text{TP0865/ECL2}}$	QYPYLTMEGKAVGGVAI	FW - AATGGAGGGAAAGGCAGTAGGCGGCGTTGCCATTTCCCCGTGTCGTCTGGTTG<br>RV - GCCTTTCCCTCCATTGTCAGGTAGGGATACTGTCCACCGCATCCCGGAATG
TP0865	ECL3	*Pf*Trx$^{\text{TP0865/ECL3}}$	GYRDSSAGGERNKKNQGGKKHVVV	FW - GGAGAGGAATAAAAAAAACCAGGGGGGTAAGAAGTCCCCGTGTCGTCTGGTTG<br>RV - TTTTTATTCCTCTCCCCGCCGGCTGAAGAATCTCCACCGCATCCCGGAATG
TP0865	ECL4	*Pf*Trx$^{\text{TP0865/ECL4}}$	VKNVGLSVEVDASNSGSSMSGGRTVHATNSS	FW - GGGATGCGGTGGATCCGTCAAAAACGTTGGACTTTCAGTTG<br>RV - GACGACACGGGGATCCACTCGAGTTTGTAGCGTGCAC
TP0865	ECL5	*Pf*Trx$^{\text{TP0865/ECL5}}$	KYNVQEFADNNRF	FW - GCAAGAATTTGCAGACAACAATAGATTCTCCCCGTGTCGTCTGGTTG<br>RV - TCTGCAAATTCTTGCACGTTGTACTTTCCACCGCATCCCGGAATG
TP0865	ECL6	*Pf*Trx$^{\text{TP0865/ECL6}}$	LTGLASDI	FW - CACCGGGCTCGCCTCAGATTCCCCGTGTCGTCTGGTTG<br>RV - GAGGCGAGCCCGGTGAGTCCACCGCATCCCGGAATG
TP0865	ECL7	*Pf*Trx$^{\text{TP0865/ECL7}}$	YESDKDE	FW - TGAGTCAGACAAGGATGAGTCCCCGTGTCGTCTGGTTG<br>RV - TCCTTGTCTGACTCATATCCACCGCATCCCGGAATG
TP0865	Hatch	*Pf*Trx$^{\text{TP0865/Hatch}}$	RTASLGAWSSQGGEVLGEVRARVPAHRRVRRAVSGTSVTPVVAMAAKTSEKQKGVGRRALSLRTGGRYEMLGLAFTALADDASFFEANAAGSAAFPY	FW - GGGATGCGGTGGATCCTCTTCGCAAGGGGGTGAAGTTCTGG<br>RV - GACGACACGGGGATCCCCTCAGCGAGAGGGCACG
***Pf*Trx**		*Pf*Trx^{Empty}	MGSSHHHHHHSSGLVPRGSHMSSGIIEYDGEIDFTKGRVVLWFSIPGCGGSPCRLVERFMTELSEYFEDIQIVHINAGKWKNIVDKFNILNVPTLVYLKDGREVGRQNLIRSKEEILKKLKELQE	FW - TGCAGGAGTAACTCGAGCACCACCACCA<br>RV - CGAGTTACTCCTGCAGCTCTTTCAGT
**FadL ECLs used for animal immunizations**		*Pf*Trx$^{\text{TP0856/ECL2}}$ No AviTag	MRMFFPESGFNFSPSTGPVCTPASNPIKKLGGLGIVNF	FW - TGCAGGAGTAACTCGAGCACCACCACCA<br>RV - CGAGTTACTCCTGCAGCTCTTTCAGT
		*Pf*Trx$^{\text{TP0856/ECL4}}$ No AviTag	LPGSPFVLCRATGEQCCKTCSGRCTGVGTCCNGEKPCCKDCDCNCPCQDEATPGSPHATDTML	
		*Pf*Trx$^{\text{TP0858/ECL2}}$ No AviTag	MFFPESGFDFSTTTEPVCTPASNPIKQRGA	
		*Pf*Trx$^{\text{TP0858/ECL4}}$ No AviTag	AATNLGLTVKVSDKIENCTSTCEKCGCCKERCCCNGKKACCKDCDCNCPCQDCNDKGTVHATDTML	
		*Pf*Trx$^{\text{TP0865/ECL3}}$ No AviTag	DSSAGGERNKKNQGGKK	
**TbpB loopless C-lobe (TbpB-LCL)**		TbpB-LCL$^{\text{TP0856/ECL2}}$	GSSSENKLTTVLDAVELTLNDKKIKNLDNFSNAAQLVVDGIMIDLAGTEFTRKFEHTMRMFFPESGFNFSPSTGPVCTPASNPIKKLGGLGIVNFTYEVEVCCSNLNYLKYGMLTRKGKQVEQSMFLQGERTDEKEIPTDQNVVYRGSWYGHIANGTSWSGNASDKEGGNRAEFTVNFADKKITGKLTAENRQAQTFTIEGMIQGNGFEGTAKTAESGFDLDQKNTTRTPKAYITDAKVKGGFYGPKAEELGGWFAYPGNAPEGKQEKATVVFGAKRQQPVQGEAAAKEAAAKGLNDIFEAQKIEWHE	synthetic gene
		TbpB-LCL$^{\text{TP0856/ECL4}}$	GSSSENKLTTVLDAVELTLNDKKIKNLDNFSNAAQLVVDGIMIDLAGTEFTRKFEHTLPGSPFVLCRATGEQCCKTCSGRCTGVGTCCNGEKPCCKDCDCNCPCQDEATPGSPHATDTMLTYEVEVCCSNLNYLKYGMLTRKGKQVEQSMFLQGERTDEKEIPTDQNVVYRGSWYGHIANGTSWSGNASDKEGGNRAEFTVNFADKKITGKLTAENRQAQTFTIEGMIQGNGFEGTAKTAESGFDLDQKNTTRTPKAYITDAKVKGGFYGPKAEELGGWFAYPGNAPEGKQEKATVVFGAKRQQPVQGEAAAKEAAAKGLNDIFEAQKIEWHE	synthetic gene
		TbpB-LCL$^{\text{TP0858/ECL2}}$	GSSSENKLTTVLDAVELTLNDKKIKNLDNFSNAAQLVVDGIMIDLAGTEFTRKFEHTMFFPESGFDFSTTTEPVCTPASNPIKQRGATYEVEVCCSNLNYLKYGMLTRKGKQVEQSMFLQGERTDEKEIPTDQNVVYRGSWYGHIANGTSWSGNASDKEGGNRAEFTVNFADKKITGKLTAENRQAQTFTIEGMIQGNGFEGTAKTAESGFDLDQKNTTRTPKAYITDAKVKGGFYGPKAEELGGWFAYPGNAPEGKQEKATVVFGAKRQQPVQGEAAAKEAAAKGLNDIFEAQKIEWHE	synthetic gene
		TbpB-LCL$^{\text{TP0858/ECL4}}$	GSSSENKLTTVLDAVELTLNDKKIKNLDNFSNAAQLVVDGIMIDLAGTEFTRKFEHTAATNLGLTVKVSDKIENCTSTCEKCGCCKERCCCNGKKACCKDCDCNCPCQDCNDKGTVHATDTMLTYEVEVCCSNLNYLKYGMLTRKGKQVEQSMFLQGERTDEKEIPTDQNVVYRGSWYGHIANGTSWSGNASDKEGGNRAEFTVNFADKKITGKLTAENRQAQTFTIEGMIQGNGFEGTAKTAESGFDLDQKNTTRTPKAYITDAKVKGGFYGPKAEELGGWFAYPGNAPEGKQEKATVVFGAKRQQPVQGEAAAKEAAAKGLNDIFEAQKIEWHE	synthetic gene
		TbpB-LCL$^{\text{TP0865/ECL3}}$	GSSSENKLTTVLDAVELTLNDKKIKNLDNFSNAAQLVVDGIMIDLAGTEFTRKFEHTDSSAGGERNKKNQGGKKTYEVEVCCSNLNYLKYGMLTRKGKQVEQSMFLQGERTDEKEIPTDQNVVYRGSWYGHIANGTSWSGNASDKEGGNRAEFTVNFADKKITGKLTAENRQAQTFTIEGMIQGNGFEGTAKTAESGFDLDQKNTTRTPKAYITDAKVKGGFYGPKAEELGGWFAYPGNAPEGKQEKATVVFGAKRQQPVQGEAAAKEAAAKGLNDIFEAQKIEWHE	synthetic gene
		TbpB-LCL^{Empty}	GSSSENKLTTVLDAVELTLNDKKIKNLDNFSNAAQLVVDGIMIDLAGTEFTRKFEHTINGKTYEVEVCCSNLNYLKYGMLTRKGKQVEQSMFLQGERTDEKEIPTDQNVVYRGSWYGHIANGTSWSGNASDKEGGNRAEFTVNFADKKITGKLTAENRQAQTFTIEGMIQGNGFEGTAKTAESGFDLDQKNTTRTPKAYITDAKVKGGFYGPKAEELGGWFAYPGNAPEGKQEKATVVFGAKRQQPVQGEAAAKEAAAKGLNDIFEAQKIEWHE	synthetic gene
		TbpB-LCL^{ECL} into pRB1B vector		FW - CGCGCGGCAGCCATATGGGCAGCAGCAGCGAAAAC<br>RV - GGTGGTGGTGCTCGAGTTATTCATGCCATTCAATTTTCTGC

Amino acid sequences of ECL boundaries predicted by trRosetta (highlighted in bold) and AlphFold3 (underlined) for all three OMP families investigated in this study. Amino acid sequences and primer pairs utilized for *Pf*Trx-scaffolded ECL constructs (ECL1-Salmon, ECL2-Blue, ECL3-Purple, ECL4-Green, ECL5-Yellow, ECL6-Cyan, ECL7-Dark Teal, and Hatch-Red).
