# Supplementary material for: Immunodominant extracellular loops of Treponema pallidum FadL outer membrane proteins elicit antibodies with opsonic and growth-inhibitory activities
Source: PLoS Pathog. 2024 Dec 23;20(12):e1012443. doi: 10.1371/journal.ppat.1012443 (PMC11761103; doi:10.1371/journal.ppat.1012443)
Supplement: S2 Table — (PDF) [file ppat.1012443.s008.pdf]

| | Attached *TPA* Rabbit Assay | | | | | | | | | | | |
|---|---|---|---|---|---|---|---|---|---|---|---|---|
| | 1% | | | | 5% | | | | 10% | | | |
| | Mean % (Range %) | $p$-value$^{\#}$ | $p$-value$^{*}$ | $p$-value$^{\blacktriangle}$ | Mean % (Range %) | $p$-value$^{\#}$ | $p$-value$^{*}$ | $p$-value$^{\blacktriangle}$ | Mean % (Range %) | $p$-value$^{\#}$ | $p$-value$^{*}$ | $p$-value$^{\blacktriangle}$ |
| **TpCM-2 medium** | 83 (81-84) | n.s | 0.0002 | < 0.0001 | 75 (71-78) | n.s | n.s | n.s | 83 (81-84) | n.s | n.s | n.s |
| **NRS** | 82 (81-82) | n.s | 0.0003 | < 0.0001 | 81 (78-83) | n.s | 0.0191 | 0.0278 | 82 (81-82) | n.s | n.s | n.s |
| **IRS 112** | 31 (26-43) | < 0.0001 | 0.0189 | n.s | 39 (30-44) | 0.0002 | n.s | n.s | 26 (13-39) | < 0.0001 | n.s | 0.0244 |
| **α-TP0856 ECL2** | 52 (45-59) | 0.0008 | | n.s | 52 (50-55) | 0.0245 | | n.s | 52 (38-66) | n.s | | n.s |
| **α-TP0856 ECL4** | 88 (87-89) | n.s | < 0.0001 | < 0.0001 | 86 (83-91) | n.s | 0.0026 | 0.0037 | 75 (69-78) | n.s | n.s | n.s |
| **α-TP0858 ECL2** | 79 (65-89) | n.s | 0.0011 | < 0.0001 | 68 (50-79) | n.s | n.s | n.s | 58 (49-69) | n.s | n.s | n.s |
| **α-TP0858 ECL4** | 74 (64-79) | n.s | 0.0189 | 0.0002 | 66 (51-89) | n.s | n.s | n.s | 67 (51-80) | n.s | n.s | n.s |
| **α-TP0865 ECL3** | 94 (92-96) | n.s | < 0.0001 | < 0.0001 | 90 (89-91) | n.s | 0.0007 | 0.0010 | 74 (66-85) | n.s | n.s | n.s |
| **α-BamA ECL4** | 43 (35-49) | < 0.0001 | n.s | | 53 (49-60) | 0.0356 | n.s | | 60 (38-76) | n.s | n.s | |
| **α-TP0751** | 84 (83-85) | n.s | < 0.0001 | < 0.0001 | 95 (92-97) | n.s | 0.0001 | 0.0002 | 70 (69-72) | n.s | n.s | n.s |
| **α-Tpp17** | 80 (78-82) | | 0.0008 | < 0.0001 | 80 (78-82) | | 0.0245 | 0.0356 | 83 (82-84) | | n.s | n.s |
| **α-*Pf*Trx** | 87 (86-88) | n.s | < 0.0001 | < 0.0001 | 93 (91-95) | n.s | 0 | 0.0003 | 91 (89-91) | n.s | 0.0071 | n.s |

Statistical analysis was done using one-way ANOVA. #: vs. α-Tpp17; *: vs. α-TP0856 ECL2; ▲: vs. α-BamA ECL4
