# Supplementary material for: Immunodominant extracellular loops of Treponema pallidum FadL outer membrane proteins elicit antibodies with opsonic and growth-inhibitory activities
Source: PLoS Pathog. 2024 Dec 23;20(12):e1012443. doi: 10.1371/journal.ppat.1012443 (PMC11761103; doi:10.1371/journal.ppat.1012443)
Supplement: S3 Table — (PDF) [file ppat.1012443.s009.pdf]

	Attached *TPA* Mouse Assay							
	5%							
	**Mean % (Range %)**	***p*-value#**	***p*-value***	**p-value△**	**p-value♠**	***p*-value★**	***p*-value✦**	***p*-value▲**
**TpCM-2 medium**	84 (83-85)	n.s	0.0008	0.0131	< 0.0001	< 0.0001	< 0.0001	< 0.0001
**NMS**	84 (82-86)	n.s	0.0008	0.0131	< 0.0001	< 0.0001	< 0.0001	< 0.0001
**MSS**	52 (49-56)	< 0.0001	n.s	0.0055	0.0036	0.0015	n.s	n.s
**α-TP0856 ECL2**	64 (54-72)	< 0.0001		n.s	< 0.0001	< 0.0001	0.0019	0.0006
**α-TP0856 ECL4**	68 (61-76)	< 0.0001	n.s		< 0.0001	< 0.0001	0.0001	< 0.0001
**α-TP0858 ECL2**	34 (34-35)	< 0.0001	< 0.0001	< 0.0001		n.s	n.s	n.s
**α-TP0858 ECL4**	33 (31-34)	< 0.0001	< 0.0001	< 0.0001	n.s		n.s	n.s
**α-TP0865 ECL3**	46 (42-48)	< 0.0001	0.0019	0.0001	n.s	n.s		n.s
**α-BamA ECL4**	45 (42-45)	< 0.0001	0.0006	< 0.0001	n.s	n.s	n.s	
**α-TP0751**	90 (84-93)	n.s	< 0.0001	0.0003	< 0.0001	< 0.0001	< 0.0001	< 0.0001
**α-Tpp17**	93 (91-94)		< 0.0001	< 0.0001	< 0.0001	< 0.0001	< 0.0001	< 0.0001
**α-*Pf*Trx**	86 (80-92)	n.s	0.0002	0.0029	< 0.0001	< 0.0001	< 0.0001	< 0.0001

Statistical analysis was done using one-way ANOVA. #: vs. α-Tpp17; *: vs. α-TP0856 ECL2; △: vs. α-TP0856 ECL4; ♠: vs. α-TP0858 ECL2; ★: vs. α-TP0858 ECL4; ✦: vs. α-TP0865 ECL3; ▲: vs. α-BamA ECL4
